# Competition for Mitogens Regulates Spermatogenic Stem Cell Homeostasis in an Open Niche

**DOI:** 10.1016/j.stem.2018.11.013

**Published:** 2019-01-03

**Authors:** Yu Kitadate, David J. Jörg, Moe Tokue, Ayumi Maruyama, Rie Ichikawa, Soken Tsuchiya, Eri Segi-Nishida, Toshinori Nakagawa, Aya Uchida, Chiharu Kimura-Yoshida, Seiya Mizuno, Fumihiro Sugiyama, Takuya Azami, Masatsugu Ema, Chiyo Noda, Satoru Kobayashi, Isao Matsuo, Yoshiakira Kanai, Takashi Nagasawa, Yukihiko Sugimoto, Satoru Takahashi, Benjamin D. Simons, Shosei Yoshida

**Affiliations:** 1 Division of Germ Cell Biology, National Institute for Basic Biology, National Institutes of Natural Sciences, 5-1 Higashiyama, Myodaiji, Okazaki 444-8787, Japan; 2 Department of Basic Biology, School of Life Science, Graduate University for Advanced Studies (Sokendai), 5-1 Higashiyama, Myodaiji, Okazaki 444-8787, Japan; 3 The Wellcome Trust/Cancer Research UK Gurdon Institute, University of Cambridge, Tennis Court Road, Cambridge CB2 1QN, UK; 4 Cavendish Laboratory, Department of Physics, University of Cambridge, J.J. Thomson Avenue, Cambridge CB3 0HE, UK; 5 Department of Pharmaceutical Biochemistry, Kumamoto University Graduate School of Pharmaceutical Sciences, Oe-Honmachi, Kumamoto 862-0973, Japan; 6 AMED-CREST, Japan Agency for Medical Research and Development, Tokyo 100-0004, Japan; 7 Department of Biological Science and Technology, Faculty of Industrial Science and Technology, Tokyo University of Science, 6-3-1 Niijuku, Katsushika-ku, Tokyo 125-8585, Japan; 8 Department of Veterinary Anatomy, The University of Tokyo, Yayoi 1-1-1, Bunkyo-ku, Tokyo 113-8657, Japan; 9 Department of Molecular Embryology, Research Institute, Osaka Women’s and Children’s Hospital, Osaka Prefectural Hospital Organization 840, Murodo-cho, Izumi, Osaka, 594-1101, Japan; 10 Laboratory Animal Resource Center, University of Tsukuba, 1-1-1 Tennodai, Tsukuba 305-8575, Japan; 11 Department of Stem Cells and Human Disease Models, Research Center for Animal Life Science, Shiga University of Medical Science, Seta, Tsukinowa-cho, Otsu, Shiga 520-2192, Japan; 12 Division of Environmental Photobiology, National Institute for Basic Biology, Okazaki 444-8585, Japan; 13 Life Science Center for Survival Dynamics, Tsukuba Advanced Research Alliance (TARA), University of Tsukuba, Tsukuba, Ibaraki 305-8577, Japan; 14 Laboratory of Stem Cell Biology and Developmental Immunology, Graduate School of Frontier Biosciences, Graduate School of Medicine, Immunology Frontier Research Center, World Premier International Research Center (WPI), Osaka University, Osaka 565-0871, Japan; 15 Department of Anatomy and Embryology, Faculty of Medicine, University of Tsukuba, 1-1-1 Tennodai, Tsukuba 305-8575, Japan; 16 Wellcome Trust-Medical Research Council Stem Cell Institute, University of Cambridge, Cambridge CB2 1QR, UK

**Keywords:** stem cells, spermatogenesis, density homeostasis, open niche, biophysical modeling, mitogen competition, fibroblast growth factors

## Abstract

In many tissues, homeostasis is maintained by physical contact between stem cells and an anatomically defined niche. However, how stem cell homeostasis is achieved in environments where cells are motile and dispersed among their progeny remains unknown. Using murine spermatogenesis as a model, we find that spermatogenic stem cell density is tightly regulated by the supply of fibroblast growth factors (FGFs) from lymphatic endothelial cells. We propose that stem cell homeostasis is achieved through competition for a limited supply of FGFs. We show that the quantitative dependence of stem cell density on FGF dosage, the biased localization of stem cells toward FGF sources, and stem cell dynamics during regeneration following injury can all be predicted and explained within the framework of a minimal theoretical model based on “mitogen competition.” We propose that this model provides a generic and robust mechanism to support stem cell homeostasis in open, or facultative, niche environments.

## Highlights

• Mouse spermatogenic stem cells (SSCs) migrate among their differentiating progeny
• Lymphatic endothelial cells near vasculature secrete FGFs that act as SSC mitogens
• SSCs tune their self-renewal and differentiation in response to FGF consumption
• Competition for limited supply of mitogen (FGFs) regulates SSC density homeostasis

## Graphical Abstract

**Figure:**
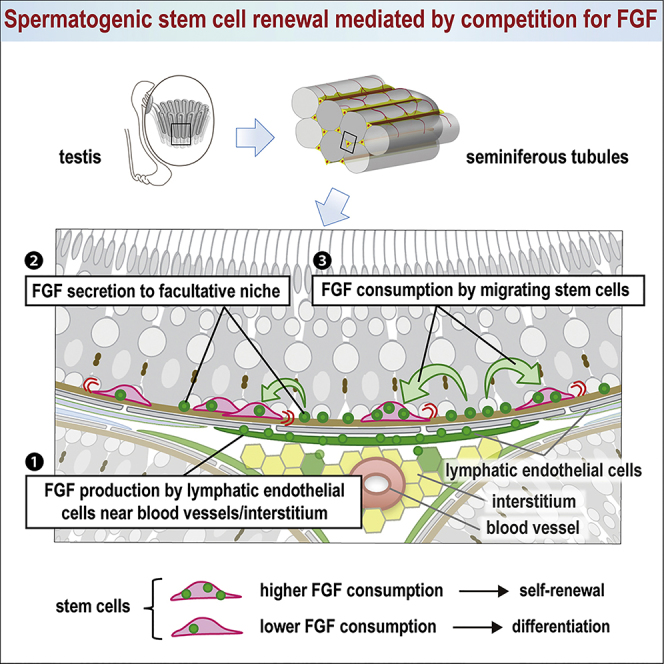

## Teaser

Focusing on mouse spermatogenesis, Kitadate et al. questioned how the stem cell pool is maintained in tissues where motile stem cells intermingle among differentiating progeny. They found that competition for a limited supply of FGFs secreted by lymphatic endothelial cells leads to self-organized density homeostasis of spermatogenic stem cells.

## Introduction

The maintenance of cycling adult tissues relies on the activity of stem cell populations. To replenish cells lost through differentiation, stem cells must balance self-renewal and differentiation (Krieger and Simons, 2015, Simons and Clevers, 2011). Such fate asymmetry may be enforced at the level of individual cell divisions or may be assigned stochastically with balance achieved only at the population level—termed “population asymmetry” (Klein and Simons, 2011). Traditionally, efforts to resolve the factors that control fate asymmetry place emphasis on short-range mitogenic and anti-differentiation signals from a definitive anatomical niche, a specialized microenvironment to which stem cells anchor, becoming physically separated from their differentiating progeny (Morrison and Spradling, 2008, Stine and Matunis, 2013, Watt and Hogan, 2000). However, in some tissues, such as mammalian blood and spermatogenesis, stem cell maintenance is thought to take place in a “facultative,” or “open,” niche (Morrison and Spradling, 2008, Stine and Matunis, 2013, Yoshida, 2018a), where stem cells are often highly motile and lie dispersed among their differentiating progeny. The question of how stem cell number is regulated in such environments is poorly understood.

In the mouse testis, the vast stem cells that support long-term homeostasis are included within the population of GFRα1^+^ spermatogonia, which comprise mononucleated (A_single_ [A_s_]) and syncytial (A_pair_ [A_pr_] and A_aligned_ [A_al_]) cells (Garbuzov et al., 2018, Hara et al., 2014, Hofmann et al., 2005). Whether the self-renewing compartment comprises all or a subset of this population remains unclear (Lord and Oatley, 2017, Yoshida, 2018b). Although some propose that long-term self-renewal potential is restricted to a small subpopulation of GFRα1^+^ A_s_ cells expressing Id4 or other markers (Chan et al., 2014, Helsel et al., 2017), others argue that the entire GFRα1^+^ population comprises a single pool in which cells interconvert between topologically distinct states of A_s_ and A_pr_/A_al_ syncytia (Hara et al., 2014). Nevertheless, during homeostasis, it is known that the size of the GFRα1^+^ pool is kept constant through population asymmetry, in which continuous and stochastic stem cell loss through differentiation is *locally* compensated by proliferation of neighbors (Hara et al., 2014, Klein et al., 2010, Klein and Simons, 2011). However, the mechanisms that ensure this balance remain undefined.

**Figure 1:**
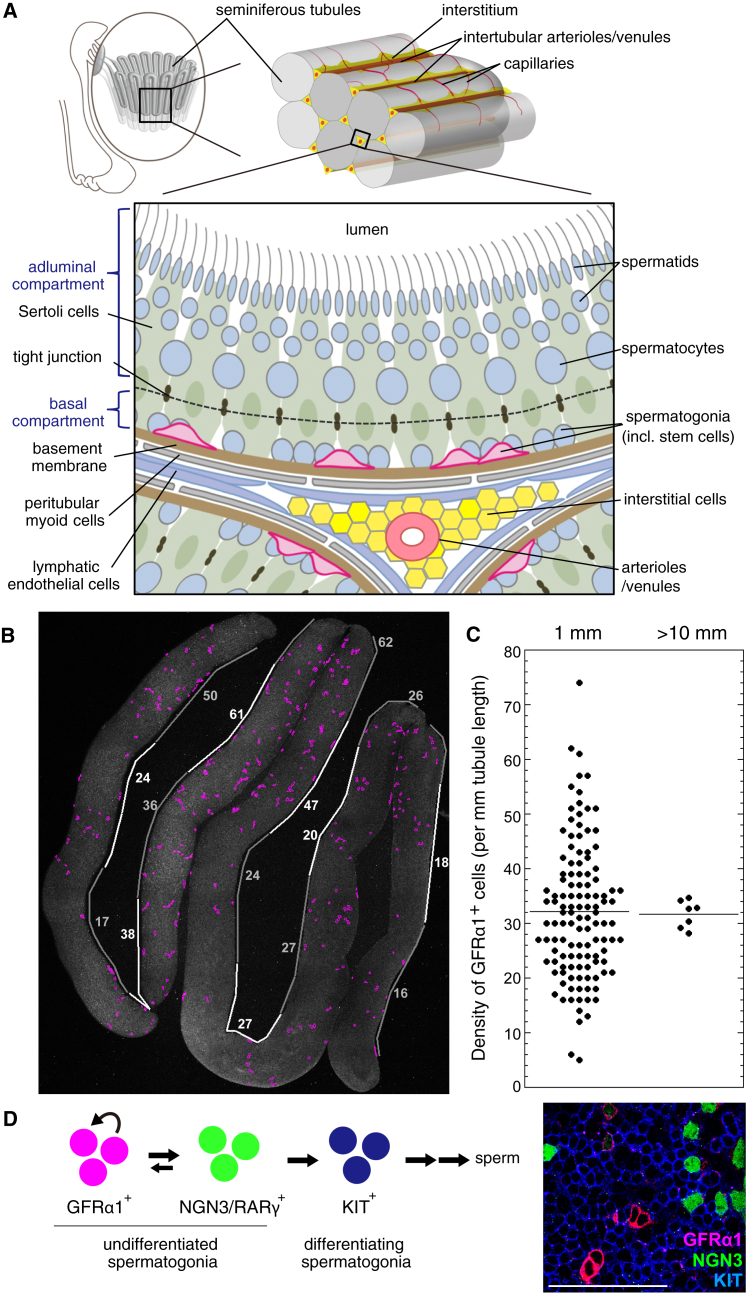
Testis Anatomy and Constant Average Density of GFRα1^+^ Stem Cells (A) Anatomy of a mouse testis and seminiferous tubules. (B) An image of a whole-mount seminiferous tubule after immunofluorescence (IF), in which positions of GFRα1^+^ cells are traced (magenta). White and gray lines alongside the tubules indicate 1-mm-long segments, containing the indicated numbers of GFRα1^+^ cells. (C) Variable numbers of GFRα1^+^ cells contained in a 1-mm-long segment (left) and the highly constant average density over long continual segments over 10 mm (right). Horizontal lines indicate the average values. (D) Hierarchy (left) and interminglement (right; a whole-mount IF of seminiferous tubules) of GFRα1^+^, NGN3/RARγ^+^, and KIT^+^ spermatogonia. Scale bar, 100 μm.

In the definitive, or closed, niche environment of *Drosophila* and *C. elegans* gonads, self-renewal-promoting signals show a restricted distribution (Spradling et al., 2011). In mouse seminiferous tubules, factors known to regulate stem cell behavior (i.e., self-renewal-promoting glial cell line-derived neurotrophic factor [GDNF], the GFRα1 ligand [Chen et al., 2016, Meng et al., 2000], and differentiation-promoting retinoic acid [RA] and Wnt) are distributed in a spatially uniform manner around the tubule, while showing periodic temporal variation in concert with the seminiferous epithelial cycle (Sato et al., 2011, Sharma and Braun, 2018, Takase and Nusse, 2016, Tokue et al., 2017, Vernet et al., 2006, Ikami et al., 2015, Oakberg, 1956, Yoshida, 2018a). However, GFRα1^+^ cells show biased localization toward the vasculature (arterioles and venules) and surrounding interstitium; yet the basis of this localization is unknown (Chiarini-Garcia et al., 2001, Hara et al., 2014, Yoshida et al., 2007). Despite such a bias, GFRα1^+^ cells are not clustered in defined regions but disperse among their differentiation-primed (NGN3^+^/RARγ^+^/Miwi2^+^) and committed (KIT^+^) progeny and show persistent and active migration on the basement membrane along and between different vasculature-associated regions (Figures 1A–1D and S1A; Ikami et al., 2015, Carrieri et al., 2017, Hara et al., 2014), emphasizing the non-canonical and open nature of the niche environment in this tissue. Strikingly, despite local fluctuations, the GFRα1^+^ cell density averaged over tubular segments is remarkably constant both spatially (Figures 1B, 1C, and S1A; Hara et al., 2014) and temporally (remaining constant even across the 8.6-day seminiferous epithelial cycle; Grasso et al., 2012, Ikami et al., 2015). This suggests that the pool size regulation of GFRα1^+^ cells is achieved in a manner that stabilizes their average density.

In this study, we report on how fibroblast growth factor (FGF) family ligands, secreted from a subset of lymphatic endothelial (LE) cells near the vascular network of arterioles and venules and accompanying interstitium, serve as critical extracellular factors that regulate GFRα1^+^ cell density homeostasis. By analyzing the population dynamics of GFRα1^+^ spermatogonia in wild-type (WT) and mutant mice, under both normal and perturbed conditions, we present evidence that competition for a limited supply of mitogens (FGFs) provides a robust and generic mechanism to support stem cell density regulation in the open niche environment of the mouse testis.

## Results

### FGF5 Expression in LE Cells Near the Vasculature and Its Mitogenic Function on GFRα1^+^ Spermatogonia

**Figure 2:**
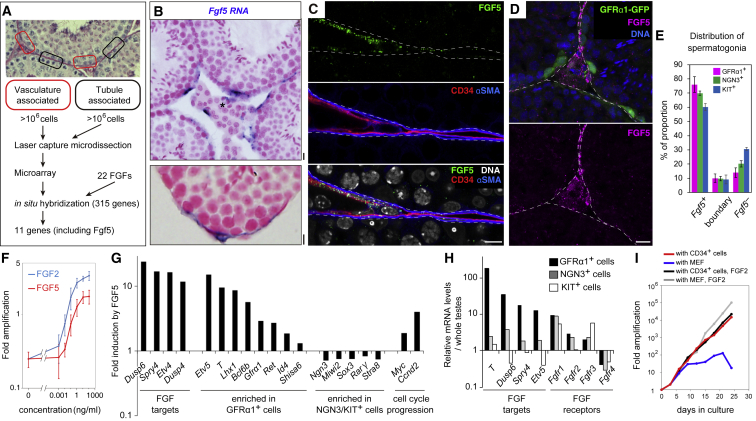
Identification, Expression, and Mitogenic Function of FGF5 (A) Outline of the screening for genes preferentially expressed in the vasculature-associated region. (B) Representative ISH images for *Fgf5* (blue) in testis sections, counterstained with nuclear fast red. Asterisk, intertubular arterioles or venules. (C) A representative IF image of a tubule periphery stained for FGF5 (green), CD34 (red), and αSMA (blue). (D) Representative image of an intertubular region of a GFRα1-GFP mouse testis stained for GFP (green), FGF5 (magenta), and DNA (blue). Scale bars, 10 μm in (B)–(D). Broken lines, outline of tubules (C and D). (E) Relationship between *Fgf5*^*+*^ area and the position of spermatogonia. Detailed data are shown in Figure S1J. (F) Mitogenic effect of FGF5 (red) or FGF2 (blue) on cultured spermatogonia. Fold increase in the number of germline stem (GS) cells cultured with indicated concentration of FGF5 for 8 days. Shown in average ± SEM (n = 3 independent experiments). (G) Effects of FGF5 on gene expression. GS cells depleted for FGF2 and GDNF for 3 days were supplemented with or without FGF5 (100 ng/mL) for 24 hr, followed by cDNA microarray analyses. (H) Gene expression of GFRα1^+^, NGN3^+^, and KIT^+^ cells *in vivo*, selected from our published cDNA microarray data of fluorescence-activated cell sorting (FACS)-sorted spermatogonial fractions, normalized to the values from whole testis (Ikami et al., 2015). (I) Amplification of GS cells co-cultured with mouse CD34^+^ testicular cells or mouse embryonic fibroblast (MEF) with or without FGF2.

As a starting point, we searched for key factors that could contribute to GFRα1^+^ cell regulation. Based on the biased localization of GFRα1^+^ cells toward the vasculature and the surrounding interstitium (Chiarini-Garcia et al., 2001, Hara et al., 2014, Yoshida et al., 2007), we compared gene expression profiles between tubule regions facing the interstitium with areas facing neighboring tubules (Hara et al., 2014; Figure 2A). These regions were collected by laser capture microdissection and processed for cDNA microarray analysis, providing 315 candidate genes enriched in vasculature-associated regions (Table S1). A second screening using *in situ* hybridization (ISH) revealed 11 genes that showed similar expression in large flattened cells covering the outer surface of the tubules near the interstitium (Figures 2B and S1B). Among these, we focused on *fibroblast growth factor 5* (*Fgf5*), because previous studies have emphasized the role of FGF signals in providing mitogenic and anti-differentiation effects on spermatogenic stem cells. This has been achieved largely *in vitro* by adding FGF2 to culture media, although the molecular identity of naturally acting FGFs *in vivo* remains elusive (Kanatsu-Shinohara et al., 2003, Kanatsu-Shinohara et al., 2014, Takashima et al., 2015).

FGF5^+^ flattened cells covered some 60% of the surface of the tubules, with a significant bias toward areas facing the interstitium (Figures S1C and S1D). Apart from a few interstitial cells, FGF5 was also immunolocalized to a subset of CD34^+^ LE cells in the two layers of peritubular cells. LE cells, sometimes termed specifically as parietal LE cells to avoid confusion with lymphatic vessel endothelial cells, are so designated because they cover the surface of lymphatic space (Clark, 1976, Kuroda et al., 2004; Figures 2C and S1E–S1G). Across the basement membrane and myoid cells, GFRα1^+^ cells showed a significant positive spatial correlation with FGF5^+^ LE cells, and NGN3^+^ cells and KIT^+^ cells showed weaker and no correlations, respectively (Figures 2D, 2E, and S1J; STAR Methods). FGF5 expression was observed throughout the seminiferous epithelial cycle (Figure S1I).

FGF5, like FGF2, promoted the proliferation of cultured GFRα1^+^ spermatogonia in a concentration-dependent manner (Figure 2F). FGF5 also led to the upregulation of genes associated with cell cycle progression (e.g., *Ccnd2* and *Myc*), the maintenance of an undifferentiated state (e.g., *Etv5*, *Id4*, *Shisa6*, *Gfrα1*, and *Ret*; Chan et al., 2014, Garbuzov et al., 2018, Meng et al., 2000, Tokue et al., 2017, Tyagi et al., 2009), and the downregulation of genes associated with differentiation (e.g., *Ngn3*, *Miwi2*, *Sox3*, *Rarγ*, and *Stra8*; Yoshida et al., 2004, Carrieri et al., 2017, Raverot et al., 2005, Gely-Pernot et al., 2012, Ikami et al., 2015, Endo et al., 2015), indicating the mitogenic and anti-differentiation effects of the factor (Figure 2G). *In vivo*, GFRα1^+^ cells expressed FGF receptors as well as high levels of genes upregulated by FGF5 *in vitro* (Figure 2H), suggesting that GFRα1^+^ cells receive the FGF5 signal. Further, in culture, CD34^+^ cells prepared according to Seandel et al., 2007, which indeed expressed *Fgf5*, supported the proliferation of GFRα1^+^spermatogonia without additional FGF in the media (Figures 2I and S1H). Together, these findings support the idea that FGF5, produced by a subset of LE cells, contributes to the regulation of GFRα1^+^ cells through mitogenic and anti-differentiation roles.

### FGFs Control GFRα1^+^ Cell Density in a Linear Dosage-Dependent Manner

**Figure 3:**
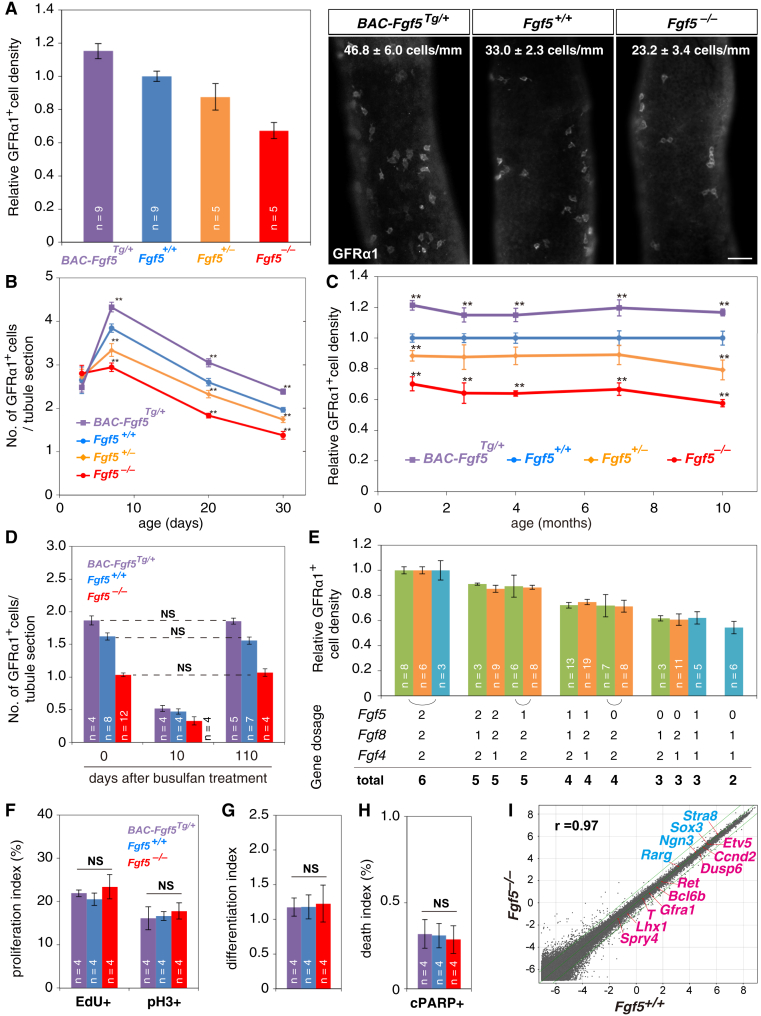
Dosage of *Fgf* Genes Quantitatively Determines the Density of GFRα1^+^ Cells (A) Average densities ± SEM of GFRα1^+^ cells and representative IF images of whole-mount seminiferous tubules for GFRα1 of 2.5-month-old mice with indicated genotypes. Scale bar, 50 μm. n, number of mice examined. (B and C) Average densities ± SEM of GFRα1^+^ cells in mice with indicated genotypes during young (B) and adult (C) ages. **p < 0.05 compared to *Fgf5^+/+^*. (D) Recovery of the GFRα1^+^ cell densities following busulfan treatment in adult mice with indicated genotypes. NS, not significant (t test). (E) Relative average densities ± SEM of GFRα1^+^ cells in mice harboring the indicated dosages of functional *Fgf5*, *Fgf8*, and *Fgf4* alleles. Results from *Fgf5-Fgf8* (green), *Fgf5-Fgf4* (orange), and *Fgf5-Fgf4-Fgf8* (cyan) intercrosses were shown separately, given their different genetic backgrounds (Figures S5J and S5K). (F–H) Indexes of proliferation (EdU^+^ and pH3^+^ fractions; F), differentiation (quantified as the RARγ^+^/KIT^−^ [≈NGN3^+^] over GFRα1^+^ cell ratio; G), and death (cPARP^+^ fraction; H) in GFRα1^+^ cells of indicated mice at 2.5 months of age. (I) Global gene expression profiles in GFRα1^+^ cells of 2.5-month-old *Fgf5*^+/+^ and *Fgf5*^−/−^ mice, indicating some positive (red) and negative (blue) FGF5 targets (Figure 2G). r, Pearson’s correlation coefficient.

We then investigated the role of FGF5 in mice carrying a null allele (*Fgf5*^*−*^) or an extra copy of bacterial artificial chromosome-mediated transgene (*BAC-Fgf5*^*Tg*^) (Khoa le et al., 2016, Mizuno et al., 2011) (Figure S2A). In *Fgf5^–/–^ ­*mutant testes, expression level of *Gdnf* did not change (Figure S2B). Similarly, the number and appearance of somatic cells, including Sertoli cells, a crucial component for stem cell regulation (Oatley et al., 2011), did not change (Figures S2C–S2E). However, the average density of GFRα1^+^ spermatogonia showed a positive and strikingly linear correlation with *Fgf5* dosage (Figure 3A). We also observed a decrease of testis weight, an increase of abnormal tubules missing one or more germ cell layers, and decrease of differentiating germ cells in accordance with the decreased *Fgf5* dose (Figures S3A–S3H).

During postnatal development, FGF5 expression was found to first accumulate in CD34^+^ cells, which cover the entire tubule surface, at around postnatal day 3 (P3), with levels becoming stronger around P7 and then localized to the vasculature-associated regions (Figure S3I). In parallel, GFRα1^+^ spermatogonia emerged postnatally by P3, both in WT and *Fgf5* mutants. By P7, their density became already correlated with *Fgf5* dosage (Figure 3B). Notably, during adulthood (up to 10 months of age), GFRα1^+^ cell density in mutants remained stable relative to WT; loss or excess of *Fgf5* caused no progressive depletion or accumulation of GFRα1^+^ cells over time (Figures 3C and S3D). Further, after an artificial reduction of the GFRα1^+^ cell pool by busulfan treatment, within 3 months, their densities recovered to their original levels specific to the respective genotypes (Figure 3D). We concluded that these mutants sustained steady-state spermatogenesis with different density set points of GFRα1^+^ cells that correlate in a manner that depends remarkably linearly on *Fgf5* dosage.

Because *Fgf5*^*−/−*^ homozygotes still maintain GFRα1^+^ cells, we examined the involvement of other FGFs and detected *Fgf4* mRNA in a pattern similar to that of *Fgf5* (Figures S4A and S4C). We also detected FGF8 protein in rat LE cells (Figures S4B and S4D). In common with *Fgf5*^*+/−*^, both *Fgf4*^*+/−*^ and *Fgf8*^*+/−*^ heterozygotes showed a proportionate reduction in GFRα1^+^ cell density, although homozygotes were embryonic lethal (Figure 3E; Meyers et al., 1998, Sun et al., 2000). Remarkably, intercross between these mutants demonstrated that GFRα1^+^ cell density also correlates linearly with the total dosage of *Fgf* genes, regardless of the combinations (Figures 3E and S4E–S4M). We concluded that multiple FGFs play key roles in the regulation of GFRα1^+^ cell density. The observed dependence of GFRα1^+^ cell density on total *Fgf* dosage indicates that FGF signaling plays a limiting role in the regulation of spermatogenic stem cell density.

### Each GFRα1^+^ Cell Receives an Unchanged Level of FGF Signal in *Fgf5* Mutants

How does FGF signaling regulate quantitatively GFRα1^+^ cell density? Given the mitogenic and differentiation-inhibiting functions of FGF (Figures 2F and 2G), we first considered whether GFRα1^+^ cells receive altered levels of FGF signal in mutants, which in turn change their fate, resulting in altered densities. Surprisingly, however, we found that the rates of proliferation, differentiation (RARγ^+^ to GFRα1^+^ cell ratio) and death of GFRα1^+^ cells were not different between *Fgf5*^*−/−*^, *BAC-Fgf5*^*Tg/+*^, and WT mice (Figures 3F–3H), indicating conserved fate behavior of GFRa1^+^ cells between mutants. Consistently, clonal fates of pulse-labeled GFRα1^+^ cells were essentially unchanged in *Fgf5*^*−/−*^ mice compared to WT (Figure S4N; Hara et al., 2014). Furthermore, gene expression profiles of GFRα1^+^ cells were highly conserved between *Fgf5*^−/−^ and WT, showing only minimal differences in FGF target genes (Figures 3I and S4O). These findings support the somewhat counterintuitive conclusion that GFRα1^+^ cells receive largely unaltered levels of FGF signal, even if the FGF dose and homeostatic GFRα1^+^ cell density change.

This unexpected observation, as well as the linear relationship between the *Fgf* dosage and GFRα1^+^ cell density, suggested that the supply of FGF may be a limiting factor that is competed for among the GFRα1^+^ cell population. In this case, the levels of FGF signal received by each GFRα1^+^ cell would be equalized among *Fgf* mutants harboring different GFRα1^+^ cell densities. To develop this hypothesis, we then examined whether GFRα1^+^ cells consume the extracellular FGF when they receive its signal *in vivo*, as this was probably the simplest form of competition consistent with the general mechanism of FGF signal reception by target cells (Ornitz and Itoh, 2015, Turner and Grose, 2010).

### GFRα1^+^ Spermatogonia Consume FGF5

**Figure 4:**
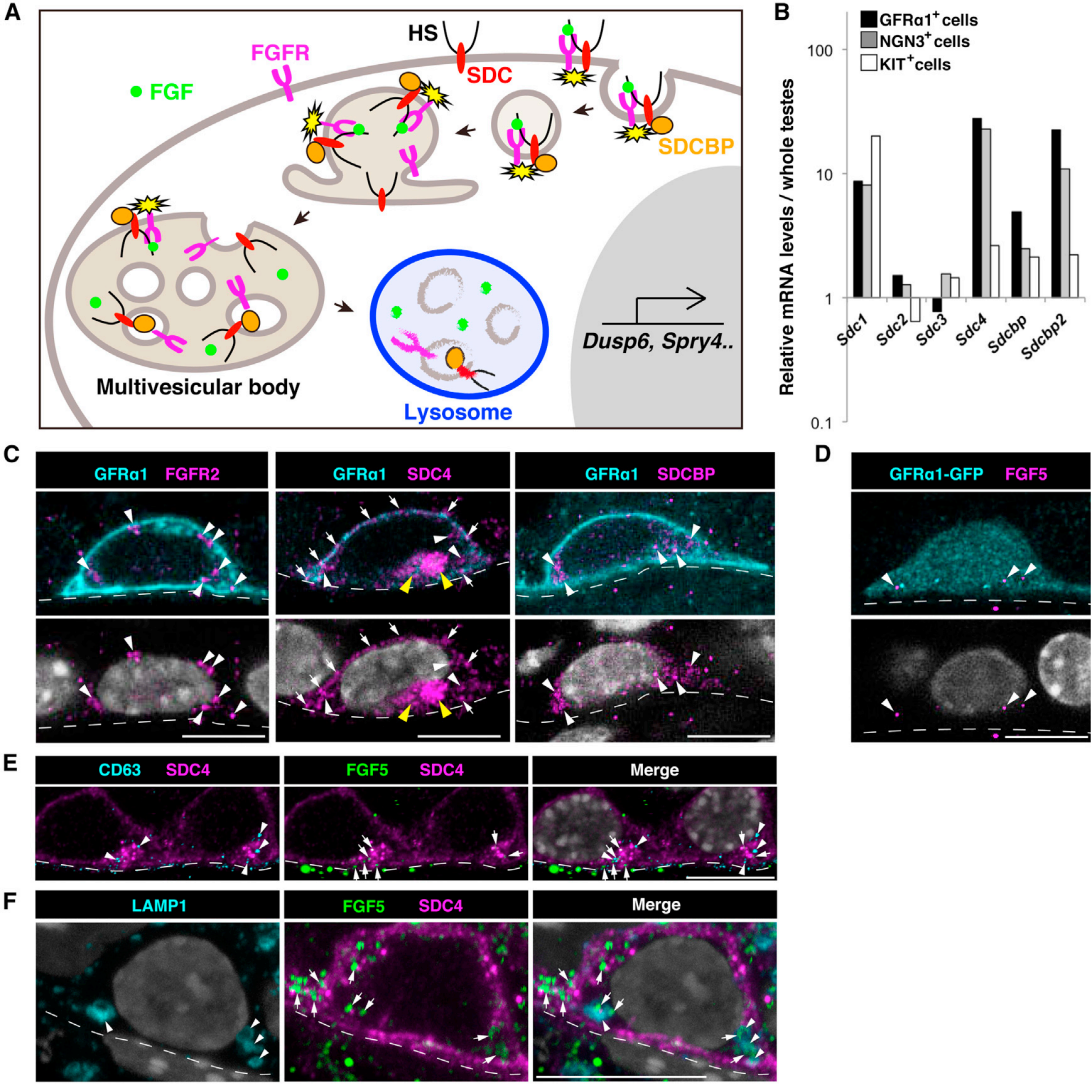
GFRα1^+^ Spermatogonia Uptake and Consume FGF5 (A) A general scheme of FGF reception on target cells, adopted from Hanson and Cashikar (2012). (Republished with permission of Annual Reviews, from Multivesicular Body Morphogenesis, Phyllis I. Hanson and Anil Cashikar, volume 28, 2012; permission conveyed through Copyright Clearance Center, Inc.) (B) Expression of genes indicated in GFRα1^+^, NGN3^+^, and KIT^+^ spermatogonial fractions, selected from published cDNA microarray data (Ikami et al., 2015), and normalized to the values from the whole testis. (C and D) Representative images of GFRα1^+^ or GFRα1-GFP^+^ (cyan) cells exhibiting the speckled cytoplasmic staining of FGFR2, SDC4, SDCBP (C), or FGF5 (D) (magenta, white arrowheads) and cell surface SDC4 staining (white arrows). Cytoplasmic SDC4 signals often form prominent clusters (yellow arrowheads). (E and F) Representative images of SDC4^+^ (magenta) cells co-stained for FGF5 (green) with CD63 (E) or LAMP1 (F; cyan). FGF5^+^ speckles (arrows) were often co-localized with CD63^+^ (arrowheads) or LAMP1^+^ (arrowheads) foci in SDC4^+^ cytoplasmic clamp. Scale bars indicate 10 µm, and the broken lines show outlines of the tubules in (C)–(F).

In general, efficient reception of FGF signal requires heparan sulfate (HS) proteoglycans (e.g., syndecans), whose HS chains bind and transfer FGFs to the receptor tyrosine kinases (FGFRs) on the target cells (Ornitz and Itoh, 2015, Turner and Grose, 2010). Reception of FGF by these molecules is accompanied by internalization with the aid of syndecan binding proteins (SDCBPs), transportation to multivesicular bodies, and degradation in lysosomes (Figure 4A; Goh and Sorkin, 2013, Hanson and Cashikar, 2012). Significant levels of mRNAs encoding these factors were detected in GFRα1^+^ cells (Figures 2H and 4B). At the protein level, FGFR2 and FGFR3 showed higher expression in GFRα1^+^ cells (Figures 4C, S5A, and S5B). Syndecan4 (SDC4) was detected predominantly on the cell surface (Figure 4C) and in multivesicular bodies (CD63^+^), which had been observed previously by EM (Chiarini-Garcia and Russell, 2002; Figures 4E, S5C, and S5D). GFRα1^+^ cells were also found to be rich in SDCBP (Figures 4C and S5E). HS chains, especially those highly sulfated, were also enriched in GFRα1^+^ cells, and HS was also found over the basement membrane (Figure S5F). Thus, GFRα1^+^ cells appeared to be well furnished with the reception machinery for FGFs.

Moreover, we detected speckled FGF5 signals inside GFRα1^+^ cells (Figure 4D). Given the undetectable levels of *Fgf5* transcripts in these cells, these signals were most likely derived from LE and interstitial cells (Figure 2B). Significant portions of FGF5 cytoplasmic signals were co-localized with SDC4 as cytoplasmic puncta, on CD63^+^ multivesicular bodies, or on LAMP1^+^ lysosomes (Figures 4E and 4F). These observations indicated that GFRα1^+^ cells consume extracellular FGF5, supporting the idea that GFRα1^+^ cells compete for FGF. Interestingly, these features were not restricted to GFRα1^+^ cells but were shared by the entire population of undifferentiated spermatogonia, including NGN3^+^ cells (Figures S5G and S5H). Together, these findings indicate that the FGF signal is active in the interstitium-proximal area, where GFRα1^+^ and NGN3^+^ undifferentiated spermatogonia may be the principal FGF target cells.

### Homeostatic Stem Cell Density Regulation Follows from a Model of “Mitogen Competition”

**Figure 5:**
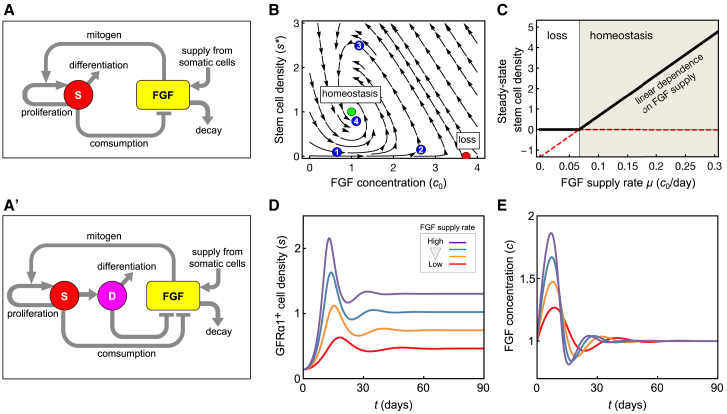
The Mitogen Competition Model (A and A′) Feedback diagrams of the “mitogen competition” model, showing the mutual regulation of the stem cell density and FGF abundance; (A) case in which only stem cells (“S”) consume FGF; (A′) case in which, in addition, the differentiation-destined progenitors (“D”) also consume FGF. (B) Phase portrait showing the dynamics of stem cell density and FGF concentration in the mitogen competition model. The system possesses two fixed points: a homeostatic state (green dot) and a loss state (red dot). For the shown parameter set, only the homeostatic state is stable, as all trajectories obtained by following the arrows converge toward this state (e.g., the trajectory indicated by numbered dots). Here, $c_{0}$ is the threshold FGF concentration at which duplication and loss by differentiation exactly balance; the steady state is set at a stem cell density $s_{*}=\left(\mu -kc_{0}\right)/k^{'}$ and $c_{*}=c_{0}$ (parameters explained in the main text). Blue dots (1–4) are provided to explain qualitatively the model dynamics (see main text). Parameters are given in Table S2. (C) Steady-state stem cell density as a function of the FGF supply rate. Above the critical supply rate, the homeostatic state is stable (shaded area) and the homeostatic stem cell density depends linearly on the FGF supply rate. Below a critical supply rate, the loss state is stable (white area) and stem cell loss is inevitable. Parameters are as in (B). (D and E) Numerical examples of the model simulation showing the GFRα1^+^ cell density (D) and tissue FGF concentration (E), starting from arbitrary initial conditions. Red, yellow, blue, and purple lines indicate different rates of FGF production ($\mu /c_{0}=0.15,0.2,0.25,0.3d^{-1}$ in this order). All other parameters are given in Table S2.

To gain deeper insight into the mechanism of density regulation, we developed a hypothesis based on the concentration-dependent mitogenic and differentiation-inhibiting activity of FGF, its supply from LE cells, and consumption by GFRα1^+^ cells (Figure 5A). To challenge this hypothesis, we developed a minimal theoretical model (Methods S1), in which stem cells (viz. GFRα1^+^ cells) are exposed to a steady supply of mitogens (viz. FGF) from microenvironment (viz. LE cells), whose consumption affects their fate behavior (viz. the probability to self-renew or differentiate). For simplicity, we first focused on the spatially averaged GFRα1^+^ cell density and mitogen concentration, returning later to consider the effect of spatial inhomogeneity of FGF production. The model is parameterized by effective rate constants that reflect the timescales of (1) stem cell proliferation and differentiation; (2) production, decay, and consumption of mitogens; and (3) the sensitivity of cell fate behavior to mitogen concentration (Figure 5A). Importantly, these population-level rate constants are not equivalent to the microscopic kinetic rate parameters; rather, they integrate the net contribution of indirect effects on mitogen consumption and processing, such as mitogen deposit to the basement membrane, mitogen diffusion, spermatogonial movement, and delays due to the successive activation of downstream targets of the FGF receptor.

Analysis of the model dynamics showed robust convergence to a homeostatic steady state, with a defined GFRα1^+^ cell density, independent of the starting condition, over a wide range of rate parameters (Figure 5B; Methods S1). Qualitatively, when the GFRα1^+^ cell density is low (cf. state “1” in Figure 5B), the net rate of FGF consumption decreases, which in turn leads to an increase of FGF concentration (cf. state “2”). This drives an increase in GFRα1^+^ cell density, as cells now tend to proliferate rather than differentiate. When the GFRα1^+^ cell density becomes too large (“3”), the opposite situation prevails, leading to a decrease of GFRα1^+^ cell density (“4”), which eventually converges to a homeostatic set point (green dot in Figure 5B). This scenario constitutes a negative feedback control on GFRα1^+^ cell density, a requisite for robust homeostatic regulation.

A key feature of this model is the emergence of a linear correlation between the homeostatic GFRα1^+^ cell density and FGF dosage (Figures 5C and 5D), as observed in *Fgf* mutants (Figures 3C and 3E). Formally, this linear dependence is given by $s^{*}=\left(\mu -kc_{0}\right)/k^{'}$, where $s^{*}$ is the homeostatic stem cell density, μ is the FGF supply rate, k is its degradation rate, $c_{0}$ is the threshold concentration at which proliferation and differentiation are balanced, and $k^{'}$ is the rate of FGF consumption by stem cells. The model also captures the counterintuitive observation that the fate behavior of GFRα1^+^ cells does not change in *Fgf* mutants (Figures 3F–3I and S4N), a consequence of the steady-state FGF concentration being always pinned at the level $c_{0}$, at which the increase (renewal) and decrease (differentiation) of GFRα1^+^ cells is balanced (Figures 5B and 5E; Methods S1). Given that GFRα1^+^ cells effectively compete with each other for the limited supply of FGFs (or mitogens more generally), we refer to this mechanism as the “mitogen competition model.”

Noting that NGN3^+^ cells may also consume FGFs (Figures S5G and S5H), we questioned whether this might affect the proposed mechanism. To this end, we extended our model to two stem and progenitor cell compartments (Figures 5A′ and S6B), which both compete for the same mitogen supply. Analysis showed that the effect of the second (progenitor) compartment is to effectively redefine the parameters of the one-component model, although the main features are not affected (Methods S1). Similarly, the key properties of the model do not rely on the premise that all GFRα1^+^ cells harbor self-renewing potential (Methods S1). Moreover, the phenomenology is not affected by the potential for “reversion” between the progenitor and stem cell compartments or by the inclusion of a stem cell death, both of which occur in homeostasis, if infrequently (Hara et al., 2014, Nakagawa et al., 2007, Nakagawa et al., 2010; Figures S6C and S6D; Methods S1). These findings emphasize the robustness of the mitogen competition mechanism in ensuring tissue-level stem cell homeostasis.

### The Mitogen Competition Model Explains the Dynamics of Recovery from Injury and the Biased Spatial Localization of Stem Cells

Having established the predictive capacity of the model under homeostatic conditions, we then questioned whether the model could predict quantitatively the dynamics of stem and progenitor cells during regeneration following injection of the cytotoxic reagent, busulfan. Analysis of the model suggested that the recovery of GFRα1^+^ cell density following a strong perturbation from its steady-state (viz. uninjured) value should be accompanied by decaying oscillations (Figures 5D and 5E). This oscillatory behavior arises due to the “inert” feedback between stem cell density and mitogen concentration (Figure 5B; Methods S1): an abrupt stem cell depletion leads to decreased FGF consumption, resulting in its accumulation; hence, stem cells now receive large amounts of FGF, leading to a bias toward proliferation beyond that experienced at homeostasis, resulting in an “overshoot” in stem cell density. This excess results in increased FGF consumption, which now lowers the FGF concentration, leading to a bias toward differentiation, pushing the density below homeostatic levels, and causing the process to restart. Indeed, the predicted density overshoot provides the means to challenge the alternative hypothesis that stem cell pool size might be determined as the maximum capacity of tissue.

**Figure 6:**
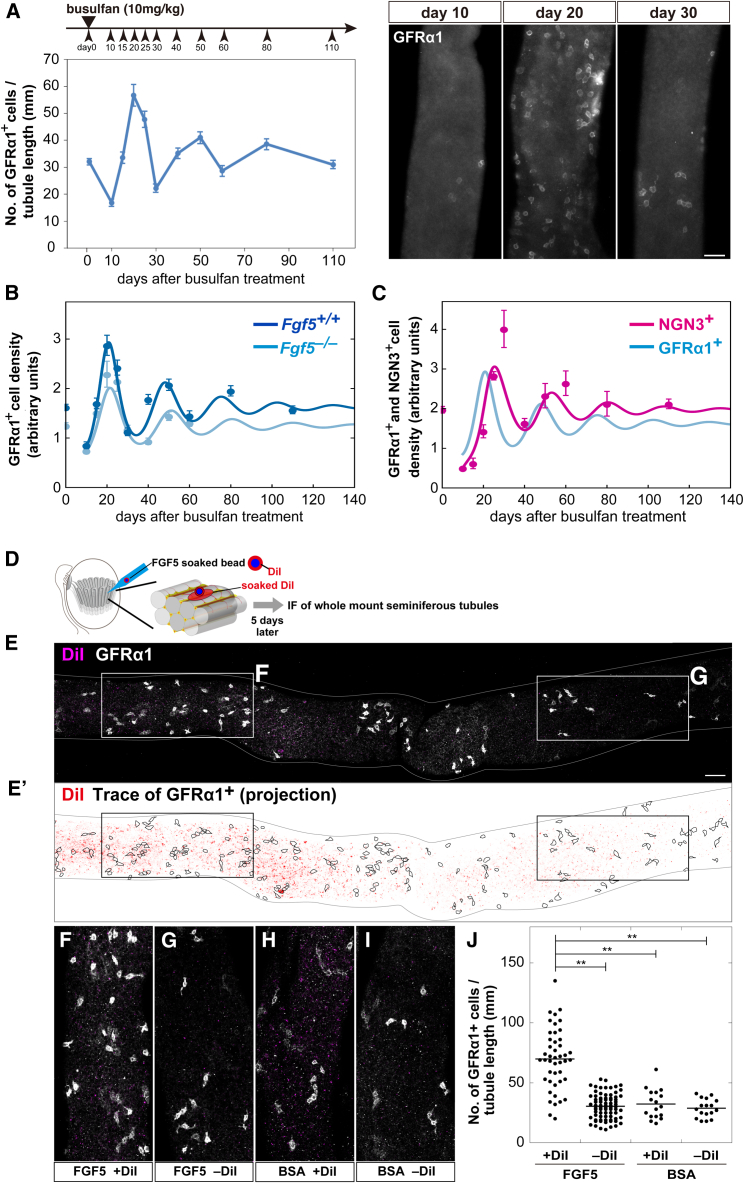
Impact of Temporal and Spatial Perturbations from Homeostasis (A) Observed kinetics of the GFRα1^+^ cell density following busulfan treatment in WT (left) and examples of IF images of whole-mount seminiferous tubules stained for GFRα1 (right) at the segments indicated by red circles in Figure S5I. (B) Model results (curves) compared to experimental measurements (dots) of the average GFRα1^+^ cell density following busulfan treatment in WT (dark blue, model fit) and *Fgf5*^−/−^ (light blue, model prediction) mice. Experimental data are rescaled from (A). (C) Model prediction (magenta curve) and experimental measurement (dots) of the average density of NGN3^+^ (in particular, RARγ^+^/KIT^−^) cells following busulfan treatment in WT. The GFRα1^+^ cell density is reproduced from (B) for comparison (blue curve). Throughout, average densities ± SEM of ≥4 testes at each data point are shown. (D) *In vivo* transplantation of DiI-labeled/FGF5-soaked beads into testicular interstitium. DiI transfers to the proximal region of the host seminiferous tubules. (E) A whole-mount image of a part of host seminiferous tubules showing the GFRα1 (white) and DiI (magenta) signals. (E’) Trace of GFRα1^+^; cells in the top (E) and bottom surfaces were projected, with enhanced DiI signal overlain (red). (F and G) Magnified images of the areas indicated in (E), representing DiI-positive (F) and negative (G) regions. (H and I) Representative images of DiI-positive (H) and negative (I) regions after transplantation of BSA-soaked beads. (J) GFRα1^+^ cell densities (numbers contained in 1-mm-long segment) in DiI-positive and negative areas after transplantation of FGF5- or BSA-soaked beads. ^∗∗^p < 0.05 compared to DiI-positive areas after transplantation of FGF5-soaked beads (t test). Scale bars, 50 μm.

To test this prediction, we examined the kinetics during recovery after busulfan treatment in WT animals and, indeed, found decaying oscillations of GFRα1^+^ cell density that converged toward the steady-state value over several months (Figure 6A) with a profile that matched quantitatively with theory (Figure 6B). Using the corresponding parameter fit, the model further predicted an altered oscillation amplitude for decreased FGF supply (Figure 6B), as well as the phase shift of oscillations of the NGN3^+^/RARγ^+^ cell density (Figure 6C), supporting the integrity of the mitogen competition model.

Finally, we questioned whether the mechanism of mitogen competition could further explain the biased localization of GFRα1^+^ (and, to a lesser extent, NGN3^+^) cells to FGF sources (Figures 2D and 2E). Indeed, an extension of the model accounting for a spatial distribution of FGF sources and spermatogonial motility (Hara et al., 2014) could predict the emergence of such a bias while preserving the global characteristics of the population-level models (Figures S6F–S6H; Methods S1). Within the framework of the model, localization of GFRα1^+^ cells to the vicinity of FGF sources arises solely from their acquired bias toward stem cell proliferation, whereas those more remote have a greater tendency to become lost through differentiation. In this regard, the mitogen competition model suggests a key role for migratory activity in combination with dynamic cell fate biases in promoting localization, and chemo-attraction may not be requisite to explain spatial biases.

Finally, to challenge the predicted causal relationship between the local FGF concentration and GFRα1^+^ cell density, we perturbed the distribution of FGF by implantation of FGF5-soaked beads alongside the seminiferous tubules for 5 days, as described previously (Figure 6D; Uchida et al., 2016). Untangled tubules were then immunostained to define the position of GFRα1^+^ cells in relation to that of the beads marked by DiI. These results showed that the GFRα1^+^ cell density was locally increased in areas proximate to FGF5-soaked beads, but not BSA-soaked beads, supporting the conclusion that FGFs locally regulate GFRα1^+^ cell density (Figures 6E–6J). The increased density of GFRα1^+^ cells in areas adjacent to the beads paralleled an increased EdU uptake (Figures S6M and S6N). Over longer time courses, the GFRα1^+^ cell density decreased, followed by an increase of GFRα1^−^ undifferentiated spermatogonia, a trend captured by theory (Figure S6M). Indeed, this increase may explain recent reports that, perhaps counterintuitively, associate FGF2 with the upregulation of RARγ and the promotion of differentiation (Masaki et al., 2018).

## Discussion

**Figure 7:**
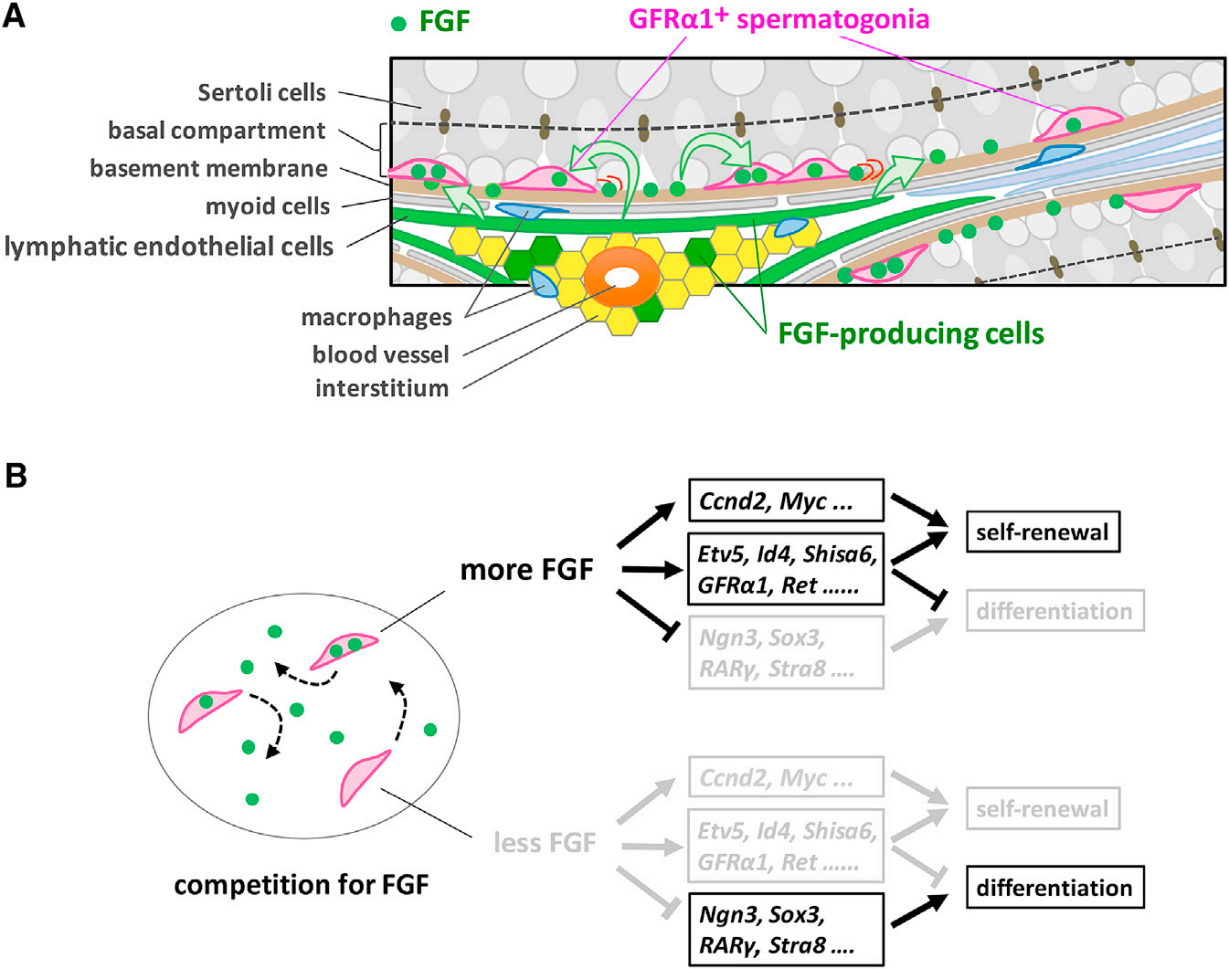
Proposed Stem Cell Regulation in Seminiferous Tubules by Mitogen Competition (A) A schematic of the microenvironment regulating GFRα1^+^ spermatogonia. In the basal compartment of seminiferous tubules, FGFs are produced and secreted by a subset of LE cells and a few interstitial cells. FGFs, which have an affinity to the basement membrane, are taken up and consumed by motile GFRα1^+^ spermatogonia and biases their fate behavior in a concentration-dependent manner (see B). This leads to higher local densities of GFRα1^+^ cells near the FGF source (near the interstitium accompanying arterioles or venules) compared to those distant from the source. (B) In the basal compartment, GFRα1^+^ cells effectively compete for a limited supply of FGF. Cells receiving larger amounts of FGF will show higher expression of cell-cycle-promoting and anti-differentiation genes and lower expression of differentiation-promoting genes, tilting their fate toward proliferation without differentiation. Cells receiving smaller amounts of FGF will show opposite patterns of target genes, tilting their fate toward differentiation.

In this study, we have targeted the mechanism of spermatogenic stem cell density homeostasis in the open, or facultative, niche environment of the basal compartment of seminiferous tubules. Our results show that the *in vivo* fate behavior of GFRα1^+^ cells is regulated by the mitogenic and anti-differentiation effects of FGFs (FGF5, 8, 4, and possibly others) released from a subset of LE, and interstitial, cells that lie in proximity to the vasculature (Figure 7A). We propose competition for mitogens as a mechanism that can explain the regulation of stem cell pool size, as well as their bias toward the mitogen source. In this framework, FGFs play a key role as fate determinants for which stem cells compete (Figure 7B). Cells receiving more FGF become biased toward proliferation over differentiation, most likely through elevated expression of cell-cycle-promoting and differentiation-inhibiting genes, as well as decreased expression of differentiation genes. In contrast, cells receiving less FGF become primed toward differentiation with opposite patterns of FGF target gene expression. We found that a minimal model of mitogen competition could account quantitatively for a variety of key properties, including the dependence of the homeostatic stem cell density on mitogen supply and the oscillatory recovery toward steady state after drug-induced perturbation. Together, these findings suggest that feedback through mitogen consumption plays a major role in density regulation of spermatogenic stem cells, although additional mechanisms of competition cannot be ruled out.

The mitogen competition mechanism does not rely on whether the stem cell compartment is “hierarchical” or whether it receives influx from a differentiating progenitor compartment via “reversion” or “dedifferentiation” (Figures S6C and S6D; Helsel et al., 2017, Yoshida, 2018b). In this context, we found that Id4, a proposed stem cell marker (Chan et al., 2014), was widely expressed across and even beyond the GFRα1^+^ cell population both at mRNA and protein levels (Figures S7A–S7C), consistent with La et al. (2018), although Id4^+/high^ cells were found to be spatially correlated with FGF5^+^ LE cells and the interstitium (Figures S7D and S7E; Yoshida, 2018a).

This study identifies FGF-producing LE cells as a key regulator of spermatogenic stem cells, which work in concert with other cells, such as Sertoli cells, myoid cells, Leydig cells, and macrophages (Chen et al., 2016, DeFalco et al., 2015, Oatley et al., 2011). It is notable that LE cells express FGF5 at uniform levels over the seminiferous epithelial cycle (Figure S1I). This contrasts GDNF, WNT, and RA signals, which show temporal oscillation in synchrony with the seminiferous cycle-related (and spatially homogeneous) gene expression of Sertoli cells (Grasso et al., 2012, Ikami et al., 2015, Sato et al., 2011, Sharma and Braun, 2018, Tokue et al., 2017, Vernet et al., 2006). Echoing this, our screening did not identify genes showing vasculature-related expression in Sertoli cells, suggesting a separation of temporal and spatial control of stem cells between Sertoli cells and FGF-producing LE cells, respectively. In future studies, it will be important to understand whether, in addition to FGFs, other signaling molecules (such as GDNF) participate directly in stem cell regulation through the same mechanism of mitogen competition and, if they do, how their function is integrated spatio-temporally with that of FGFs.

In addition to FGF5, 8, and 4, expression of FGF2 has been reported in the testis, although it was not detected in our ISH (Mullaney and Skinner, 1992, Smith et al., 1989). Given the undetectable mRNA level (Figures S4P and S4Q) and the reported nuclear localization of the protein in undifferentiated spermatogonia (Gonzalez-Herrera et al., 2006), GFRα1^+^ cells may also uptake exogenously supplied FGF2, which may play similar roles in stem cell regulation to the aforementioned FGF members.

Based on these findings, it is instructive to contrast the mechanistic basis of stem cell regulation in systems reliant on an *open*, or *facultative*, niche versus those involving a *closed*, or *definitive*, niche. In definitive niche-supported tissues, stem cells are gathered to a restricted region where mitogens are concentrated so that physical access determines stem cell pool size (Kitadate et al., 2007, Spradling et al., 2011). In contrast, in the open environment of the seminiferous tubules, stem cells are not tightly linked spatially with the source of mitogen (FGFs) but are dispersed among their differentiating progeny. Our results show that competition for mitogens, released by somatic niche cells, allows stem cells to “sense” their local density and adjust their fate bias in response, which provides a mechanistic basis to understand the dynamics of population asymmetry (Hara et al., 2014, Klein et al., 2010, Nakagawa et al., 2007).

A key element characterizing niche types is the effective range of niche-derived factors (Inaba et al., 2015). Indeed, in *Drosophila* testes and ovaries, diffusion of niche factors (e.g., bone morphogenetic protein [BMP]) is limited through their binding to heparansulfate proteoglycans (HSPGs) so that it only affects the cells next to the hub (Chen et al., 2011, Nakato and Li, 2016). In the open niche environment of mouse testis, diffusion alone may not explain the long-range effect of FGFs, because free ligands are likely diluted out quickly from the basal compartment by the systemic extravascular circulation. Rather, given the affinity with HSPGs, FGFs are expected to be immobilized (and concentrated) on the HS-rich basement membrane (Figure S5F). However, by harboring abundant HSPGs (e.g., Sdc4) and highly sulfated HS, GFRα1^+^ and NGN3^+^ spermatogonia should have a high affinity for FGFs (Figures 4C, S5D, S5F, and S5H). By up-taking FGFs from the basement membrane, the motility of spermatogonia provides a mechanism to enhance the effective range of FGFs into areas distant from the FGF source. Indeed, such “passenger diffusion” associated with the stem cell motility may underlie the long-range effect of niche factors in other open niche-supported systems.

In summary, we have shown how mitogen competition provides a basis to regulate stem cell density regulation in an open niche environment. Such behavior constitutes a novel form of “quorum sensing,” reminiscent of that exploited by bacterial populations (Miller and Bassler, 2001) and ecological systems, as it enables cells to respond to changes in the local density of neighbors through the amount of secreted factors. However, in contrast to mechanisms reliant on competition for nutrients, which lead to starvation of excess populations, here, it is the “priming” for different fate outcomes (viz. duplication versus differentiation) that leads to an effective population size control. Hence, rather than playing the role of a finite energy supply, the resource that is competed for (the mitogen) exerts a fate control. Homeostasis through mitogen competition also shares similarities with the mutual proliferative regulation of different cell types by secreted growth factors, as recently reported for fibroblasts and macrophages (Adler et al., 2018, Zhou et al., 2018); however, in the seminiferous tubules, the LE cells provide a constant supply of the signaling environment for stem cells, enabling them to restore and maintain homeostasis, even when perturbed strongly from steady state by crisis or injury.

Finally, based on these findings, it is useful to reflect on whether mitogen competition may be involved in the mechanism of tissue stem cell regulation in other contexts. In the canonical “definitive niche” environment of the *Drosophila* testis, physical contact of germline stem cells to a cluster of somatic hub cells provides both local cues that orient cell division perpendicular to the hub and access to signaling factors that maintain stem cell competence; together, these influences promote the asymmetric fate of mitotic sisters based on their proximity to the hub (Spradling et al., 2011). However, live-imaging assays show that a fraction of sister pairs undergo symmetric differentiation or symmetric self-renewal in a locally coordinated manner so that, through a local repositioning on the hub, the number of stem cells that maintain access to niche-supporting signals remains constant (Sheng and Matunis, 2011). Such stem cell renewal mechanisms may represent an extreme limit of the mitogen competition paradigm, where the extent of “mitogen” localization and degree of stem cell motility are limited. From this perspective, the functional behavior of the open (facultative) and closed (definitive) niche might not be altogether distinct. Rather, they may represent the two extremes of a continuum, in which the mechanism of mitogen competition provides a unifying framework. Whether the mechanism of mitogen competition can indeed serve as a basis to explain stem cell pool regulation in a wide variety of niche environments warrants future in-depth investigation.

## STAR★Methods

### Key Resources Table

**Table:** 

REAGENT or RESOURCE	SOURCE	IDENTIFIER
**Antibodies**		

Goat polyclonal anti-GFRα1 (used at 1:1000)	R&D	Cat#AF560, RRID: AB_2110307, Lot 0411081
Goat polyclonal anti-FGF5 (used at 1:1000)	Santa Cruz	Cat#sc-1363, RRID: AB_2102680, Lot C1810
Rat monoclonal anti-CD34 (RAM34) (used at 1:200)	eBioscience	Cat#14-0341-82, RRID: AB_467210, Lot E019241
Mouse monoclonal anti-αSMA (1A4) (used at 1:200)	Sigma-Aldrich	Cat#A5228, RRID: AB_262054, Lot 128K4843
Rabbit polyclonal anti-GFP (used at 1:400)	Thermo Fisher	Cat#A-11122, RRID: AB_221569, Lot 1828014
Rat monoclonal anti-GFP (used at 1:300)	Nacalai Tesque	Cat# 04404-84, RRID: AB_10013361
Rabbit monoclonal anti-RARγ (used at 1:200)	Cell Signaling Technology	Cat#8965, Lot 1
Goat polyclonal anti-c-Kit (used at 1:400)	R&D	Cat#AF1356, RRID: AB_354750
Rat monoclonal anti-c-Kit (used at 1:200)	BD	Cat#553355, RRID: AB_394806
Rabbit polyclonal anti-SOX9 (H-90) (used at 1:200)	Santa Cruz	Cat#sc-20095, RRID: AB_661282, Lot E1412
Rabbit polyclonal anti-CSF1R (used at 1:200)	abcam	Cat#ab183316
Rabbit monoclonal anti-StAR (used at 1:200)	Cell Signaling Technology	Cat#8449, RRID: AB_10889737, Lot 1
Rabbit polyclonal anti-phospho-Histone H3 (Ser10) (used at 1:300)	Millipore	Cat#06-570, RRID: AB_310177, Lot 2664259
Rabbit polyclonal anti-Cleaved PARP (D214) (used at 1:200)	Cell Signaling Technology	Cat#9544, RRID: AB_2160724, Lot 4
Mouse monoclonal anti-FGF8 (used at 1:1000)	Kyowa Hakko Kirin	Cat#KM1334, Lot KM1334-2
Rabbit polyclonal anit-FGFR2 (used at 1:200)	abcam	Cat#ab10648, RRID: AB_297369, Lot GR19748-1
Rabbit polyclonal anti-SDC4 (Syndecan4) (used at 1:2000)	abcam	Cat#ab24511, RRID: AB_448112, Lot GR5827-3
Rabbit polyclonal anti-SDCBP (Syntenin) (used at 1:2000)	abcam	Cat#ab19903, RRID: AB_445200, Lot GR592112-1
Rat monoclonal anti-CD63 (NVG-2) (used at 1:200)	BD	Cat#564221, Lot 4076990
Rat monoclonal anti-LAMP1 (1D4B) (used at 1:200)	Santa Cruz	Cat#sc-19992, RRID: AB_2134495, Lot L2313
Rabbit polyclonal anit-FGFR3 (used at 1:200)	abcam	Cat#ab10651, RRID: AB_297372, Lot 888076
Rabbit monoclonal anit-Id4 (used at 1:2000)	Cal Bioreagents	Cat#M106, RRID: AB_1151797
Rat monoclonal anti-ECAD (used at 1:2000)	TaKaRa	Cat#M108
Rat monoclonal anti-CD9 conjugated by Alexa Fluor 647 (used at 1:2000)	Biolegend	Cat#124809, RRID: AB_1279319
Rat monoclonal anti-c-Kit conjugated by PE/Cy7 (used at 1:2000)	Biolegend	Cat#135111, RRID: AB_2131136
Rat monoclonal anti-ECAD conjugated by PE (used at 1:2000)	This study	N/A
Mouse monoclonal anti-NAH46 conjugated by Alexa Fluor 647	This study	N/A
Mouse monoclonal anti-HepSS1 conjugated by Alexa Fluor 488	This study	N/A

**Chemicals, Peptides, and Recombinant Proteins**		

Recombinant Human FGF-5	R&D	Cat#237-F5, Lot GQ2312021
Recombinant Human FGF-basic	PeproTech	Cat#100-18B, Lot 121008
Bovine albumin	MP Biomedicals	Cat#810661
Fluoro-KEEPER Antifade Reagent	Nacalai tesque	Cat#12593-64
Hoechst33342	Thermo Fisher	Cat#H3570, Lot 23363W
Bouin’s solution	MUTO PURE CHEMICALS	Cat#3314-2, Lot 170228
Schiff reagent	Wako	Cat#193-08445, Lot LKP7191
Hematoxylin	MUTO PURE CHEMICALS	Cat#2104-2, Lot 160107
Eosin	Wako	Cat#051-06495, Lot LKQ2516
Vybrant CM-DiI cell-labeling solution	Thermo Fisher	Cat#V-22888
Affi-Gel Blue Media	BIO-RAD	Cat#153-7302, Lot 64035867
Laminin, mouse	BD	Cat#354232, Lot A1741

**Critical Commercial Assays**		

Click-iT EdU Alexa Fluor 594 or 647 Imaging Kit	Thermo Fisher	Cat#C10339 or C10640
HistoGene LCM Frozen Section Staining Kit	Thermo Fisher	Cat#KIT0401
RNeasy micro kit	QIAGEN	Cat#74004
DIG RNA Labeling Kit	Roche	Cat#1175025
Superscript III first strand synthesis system	Thermo Fisher	Cat#18080051
THUNDERBIRD SYBR qPCR Mix	TOYOBO	Cat#QPS-201
RNAscope Fluorescent Multiplex Detection Reagents	Advanced Cell Diagnostics	REF 320851, Lot 15127A
Lipofectamine 3000 Transfection Reagent	Thermo Fisher	Cat#L3000-015
Dual-Luciferase Reporter Assay System	Promega	Cat#E1960, Lot 0000144524

**Deposited Data**		

Microarray data	This study	GEO: GSE118846

**Experimental Models: Organisms/Strains**		

Mouse: *Fgf5*^*–*^ (*Fgf5*^*go-moja*^*/Utr*):C57BL6J	(Mizuno et al., 2011)	N/A
Mouse: *BAC-Fgf5*:C57BL6J	(Khoa le et al., 2016)	N/A
Mouse: *Fgf4*^*flox*^	(Sun et al., 2000)	N/A
Mouse: *Fgf8*^*–*^:CD-1	(Meyers et al., 1998)	N/A
Mouse: *Ngn3-Cre*:C57BL6J	(Yoshida et al., 2004)	N/A
Mouse: *Gfrα1-GFP*:C57BL6J	(Uesaka et al., 2007)	N/A
Mouse: *Gfrα1-CreER^T2^*:C57BL6J	(Uesaka et al., 2007)	N/A
Mouse: *CAG-CAT-GFP*:C57BL6J	(Kawamoto et al., 2000)	N/A
Mouse: *Ngn3-GFP*:C57BL6J	(Yoshida et al., 2004)	N/A

**Oligonucleotides**		

Primers for qRT-PCR, Table S3		N/A
Probe-Mm-Fgf5	Advanced Cell Diagnostics	P/N 417091, L/N 14070A
Probe-Mm-CD34-C2	Advanced Cell Diagnostics	P/N 319161-C2, L/N 14062A
Probe-Mm-Des-C3	Advanced Cell Diagnostics	P/N 407921-C3, L/N 14070A

**Recombinant DNA**		

FANTOM clones, Table S1	DNAFORM	N/A

### Contact for Reagent and Resource Sharing

Further information and requests for resources and reagents should be directed to and will be fulfilled by the Lead Contact, Shosei Yoshida (shosei@nibb.ac.jp).

### Experimental Model and Subject Details

#### Animals

The following mice were as previously described: *Fgf5*^*–*^ (*Fgf5*^*go-moja*^*/Utr*), *BAC-Fgf5, Fgf4*^*flox*^*, Fgf8*^*–*^*, Ngn3-Cre, Gfrα1-GFP,* and *Ngn3-GFP.* The background of *Fgf5*^*–*^*, Gfrα1-GFP, Ngn3-GFP, BAC-Fgf5,* and the control wild-type mice was C57BL/6 (Japan SLC, Japan CLEA), while *Fgf8*^*–*^ mice was maintained in a CD-1 background. *Fgf4* and *Fgf8* mutants were obtained from the Mutant Mouse Regional Resource Centers. Intercross between *Fgf5* and *Fgf4* or *Fgf8* mutants were done following the mating scheme (Figures S5J and S5K). Busulfan (10 mg/kg) was intraperitoneally injected to adult mice (2.5–4 months old) as described previously. All animal experiments were conducted with the approval of The Institutional Animal Care and Use Committee of National Institutes of Natural Sciences, or institutional committees for animal and recombinant DNA experiments at the Research Institute, Osaka Women’s and Children’s Hospital.

### Method Details

#### Immunofluorescence (IF)

Whole-mount IF of seminiferous tubules and IF on testis cryosections (10μm-thick) were performed as previously described (Hara et al., 2014, Nakagawa et al., 2010, Tokue et al., 2017). The following antibodies were used: anti-GFRα1, anti-FGF5, anti-CD34, anti-αSMA, anti-GFP, anti-RARγ, anti-c-Kit, anti-SOX9, anti-CSF1R, anti-StAR, anti-pH3, anti-Cleaved PARP, anti-FGF8, anit-FGFR2, anti-SDC4, anti-SDCBP, anti-CD63, anti-LAMP1, anti-FGFR3, anti-ID4. All secondary antibodies were Alexa Fluor conjugated from Life Technologies and used at 1:300 dilutions. Anti-NAH46 and anti-HepSS-1 antibodies (Seikagaku) were directly labeled with Alexa Fluor 488 5-SDP ester (Molecular Probes, A30052) and Alexa Fluor 647 NHS ester (Molecular Probes, A20006), respectively, as follows. Conjugation reaction was performed by mixing antibody solution (1.0 mg/ml in PBS) and 1/50 volume of the reactive dye (10 mg/ml) and incubating for 1.5 h at room temperature under dark conditions. Unconjugated dye was removed by gel filtration using Bio-Spin 6 Tris Columns (#732-6227; Bio-Rad) to collect the labeled antibody as a flow-through fraction. The nuclei were stained with hoechst33342 (Life Technologies). Slides were mounted in Fluoro-KEEPER Antifade Reagent (Nacalai). Observations and measurements were performed using an Olympus BX51 upright fluorescence microscope equipped with a DP72 CCD camera, a Nikon A1r confocal system, or a Leica TCS SP8 confocal system.

#### Clonal fate analysis of GFRα1^+^ cells

*Fgf5*^*+/+*^ or *Fgf5*^*–/–*^*; GFRα1-CreER^T2^; CAG-CAT-GFP* mice were injected intraperitoneally with 0.25 mg of 4-hydroxytamoxifen per individual (sigma). After the tamoxifen treatment, the testes were removed and analyzed by IF, as described previously (Hara et al., 2014).

#### Bead preparation and transplantation

Affi-Gel blue beads (Bio-Rad) were soaked in a solution of recombinant FGF5 proteins (0.1 mg/ml) or 0.1% BSA (bovine serum albumin; 0.1 mg/ml) for 1 h at room temperature according to (Uchida et al., 2016). To mark the tubular wall adjacent to the transplanted beads, some beads were immersed in DiI (0.83 mg/ml; Thermo Fisher Scientific) solution for 15 min. For transplantation, the soaked beads were transplanted into the testicular interstitium (1 or 2 beads [one per site] were separated with appropriate intervals) via vitrified micro-capillary under a dissecting microscope.

#### Laser capture microdissection

Freshly isolated testes from 8 weeks-old C57BL/6 mice were cryosectioned with 7μm-thick sections, placed on slides, fixed, and stained with HistoGene before collection of the areas of interest using PixCell IIe (ArcturusXT), according to the manufactures’ protocol. The obtained tissue fragments were proceeded for cDNA microarray gene expression analysis.

#### cDNA microarray gene expression analysis

From tissue fragments collected by laser microdissection, RNA was purified using RNeasy micro kit (QIAGEN) and processed for two-round amplification to prepare fluorescence-labeled probes as described. Briefly, in the first round, RNA samples were reverse-transcribed with T7-(dT)_24_ primer and made double-stranded, followed by cRNA synthesis using MEGAscript T7 kit (Ambion). After quality checked with an Agilent 2100 Bioanalyzer, cRNA was reverse-transcribed into ds-cDNA, and subjected to Cy3-labeled cRNA synthesis (Agilent Technology) by T7 reaction. From other materials (i.e., tissues, sorted cells, or cultured cells), RNA purification and preparation of Cy3-labeled cRNA probes were performed as described. Hybridization, scanning and data analysis were done as described. Briefly, Cy3-labeled probes were fragmented and hybridized to an Agilent whole mouse genome 4x44K or 8X60K array (Agilent Technology). Then, the image data was obtained using a G2505C scanner (Agilent Technology), analyzed using a Gene Spring software (Silicon Genetics). Data correction was performed with the threshold raw signals set to 1.0, percent shift to the 75th percentile as normalization algorithm, and no baseline transformation.

#### *in situ* hybridization (ISH) of histological sections

315 genes that showed high enrichments to vasculature-associated regions over tubule-bounding regions were selected based on the microarray data above. dsDNA fragments containing full length sequences of these transcripts were amplified from FANTOM clones (DNAFORM) using the primers of M13_Forward: CGACGTTGTAAAACGACGGCCAGTG and M13_Reverse: AGCGGATAACAATTTCACACAGGAAAC. Then, digoxigenin-labeled antisense RNA probes were synthesized using DIG RNA Labeling Kit (Roche) and processed for *in situ* hybridization on paraffin embedded sections, according to a protocol described previously (Yoshida et al., 2001).

#### ISH of dispersed testicular cells

ISH of dispersed single cells was carried out using an RNAscope Fluorescent Multiplex Kit (Advanced Cell Diagnostics). Freshly isolated testis from 8 weeks-old C57BL/6 mice were processed to generate cell suspensions of the interstitial cells and the peritubular cells on the seminiferous tubules by pipetting. The cell suspension was applied to MAS-GP-coated slide glass (Matsunami) and employed for detection of *Fgf5, Cd34* and *Des* RNA using each specific target probe and HybEZ Hybridization system (Advanced Cell Diagnostics).

#### RT-qPCR

Total RNA was extracted form samples (tissues, sorted cell or cultured cells) using RNeasy kits (QIAGEN), reverse-transcribed using Superscript III first strand synthesis kit (Life Technologies), and processed for RT-qPCR using a LightCycler 480 system (Roche) with gene-specific primers (Table S3).

#### FACS

For microarray analysis, GFRα1^+^, NGN3^+^ and KIT^+^ cell fractions were sorted by FACS, as described previously (Ikami et al., 2015, Tokue et al., 2017). The GFRα1^+^ fraction was collected from *Gfrα1-GFP* mice as the GFP^+^ fraction, and the NGN3^+^ and KIT^+^ fractions were collected from *Ngn3-GFP* mice as GFP^+^/KIT^–^ and GFP^+^/KIT^+^ fractions, respectively, using an EPICS ALTRA instrument (Beckman Coulter). The data of FACS and the microarray were partly published previously (Ikami et al., 2015, Tokue et al., 2017), with the data-set deposited in the GEO (GSE75532). In Figure 3I, GFRα1^+^ cells were collected from 2.5 months-old-adult *Gfrα1-GFP* mice in wild-type and *Fgf5*^*–/–*^ backgrounds in the same manner to (Ikami et al., 2015, Tokue et al., 2017). In Figure S7, spermatogonial fractions were sorted from *Gfr*α*1-GFP* mouse testicular cell suspension, based on CD9, ECAD, KIT and GFP staining, using a FACSAria II cell sorter (BD Biosciences). Anti-ECAD antibodies were conjugated with PE by R-Phycoerythrin AffiniPure Fab Fragment Goat Anti-Rat IgG (Jackson ImmunoResearch).

#### *In vitro* culture of spermatogonia

Germline Stem (GS) cells derived from the C57BL/6 x ICR intercrossed mice were maintained according to (Tokue et al., 2017). For the quantification of mitogenic effect of FGF2 or FGF5, 2 × 10^4^ GS cells per well of 12-well plate were cultured in the respective concentration of FGF2 or FGF5 in supplement with 10 ng/ml GDNF. After 8 days, the cell numbers were counted (n = 3 independent experiments). For the gene expression analysis, GS cells were depleted for FGF2 and GDNF for 3 days, and then supplemented with or without FGF5 (100 ng/ml) for 24 hours, followed by cDNA microarray analyses. For the co-culture with CD34^+^ cells or MEF, GS cells were cultured in supplement with GDNF, and with or without FGF2. The passage was performed every 6 days.

#### Culture of CD34^+^ cells

Primary testicular cells expressing CD34 were prepared from 8 wk-old mice referring to (Seandel et al., 2007), which designated these cells as mouse testicular stromal, or MTS, cells. Seminiferous tubules were collected from detunicated testes and minced. The tissue was washed and then enzymatically dissociated with agitation at 37 °C in a buffer containing collagenase, hyaluronidase, and DNase I. The resultant cell suspension (non-filtered) was collected, plated in dishes coated with gelatin in a 50:50 mixture of alpha MEM/StemPro-34 (Thermo Fisher Scientific) supplemented with 20% FBS and expanded over two to five passages. Cells were then cryopreserved or plated in 12-well plates coated with gelatin, and treated with mitomycin-C for 3 h, before use for co-culture with GS cells.

#### Luciferase assay

Transient transfection of GS cells cultured in 48-well plates coated with laminin (BD Biosciences) was performed using Lipofectamine-3000 (Thermo Fisher Scientific), and the culture medium was changed after 24h. Analyses were performed 24h post-medium change with or without FGF2. The luciferase activity of cell lysates was measured using a Dual-Luciferase assay system and a GloMax 20/20n luminometer (Promega). The activity of the firefly luciferase reporter pGL4-*Ngn3* was normalized to that of Renilla luciferase expressed from co-transfected pGL4.7 plasmid as described (Tokue et al., 2017).

#### Copy number determination of the BAC transgene

Genomic DNA was extracted from tail clips of WT and mutant mice according to a standard protocol, including phenol-chloroform extraction after lysis in buffers containing Proteinase K. To quantify the BAC transgene including the *Fgf5* gene (Figure S2A), the real time TaqMan PCR method using universal Probe Library probes (Roche) and gene-specific primers was used (Table S3). Copy number of *Fgf5* was determined using *Ppia* as a standard.

### Quantification and Statistical Analysis

#### Histology, evaluation of degenerating tubules, and measurement of the tubule area

Testes of WT and mutant mice were fixed with Bouin’s fixative and processed for paraffin-embedded section preparation (7μm thick) and hematoxylin and eosin staining, according to standard procedures. The percentage of degenerating seminiferous tubules was calculated based on the cross sections of seminiferous tubules (n > 200) that appeared on one transverse section for each testis. In normal (WT) mouse testes, four generations of germ cells, each synchronously progressing through spermatogenesis, form cellular associations of fixed composition (called seminiferous epithelial stages). Chronological sequence of these stages represents the periodic change of seminiferous epithelium, known as “seminiferous epithelium cycle.” In the testes of *Fgf* mutants, a few tubule cross-sections lacked one or more out of the four germ cell layers, which was defined as “degenerative tubules” in this study. The area of the cross sections of seminiferous tubules were measured (n = 255 and 195 tubule sections in WT and *Fgf5*^*–/–*^ mice, respectively). The round shape tubule cross sections were photographed under bright-field illumination, then measured the areas by a CellSens Standard software of an Olympus BX51 microscope.

#### Counting the density of GFRα1^+^ or RARγ^+^ cells

The densities of GFRα1^+^ or RARγ^+^ cells were measured on immunofluorescenced whole mount seminiferous tubules or cryo-sections of the testes. Figures 1C, 3A, right, 6A, 6B, 6J, S2E, S6M, and S6N were counted by the whole mount seminiferous tubules. For counting the cell density in 1 mm tubule length, the 1mm tubule length was measured, then the GFRa1^+^ cell number was counted by an Olympus BX51 fluorescence microscope equipped with a DP72 CCD camera or a Leica TCS SP8 confocal system. For counting the GFRα1^+^ cell density in > 10 mm tubule length, the GFRα1^+^ cell number in long continual segments over 10 mm was counted. The total length of counted tubule segments were 105 mm (Figure 1C), 140 mm of *BAC-Fgf5*^*Tg/+*^, 78 mm of *Fgf5*^*+/+*^, 94 mm of *Fgf5*^*–/–*^ mice (Figure 3A right), 122 mm (day10), 105 mm (day15), 94 mm (day20), 90 mm (day25), 160 mm (day30), 103 mm (day40), 100 mm (day50), 90 mm (day60), 100 mm (day80), 73 mm (day110) of WT mice (Figure 6A), and 61 mm (day0), 106 mm (day10), 116 mm (day15), 86 mm (day20), 91 mm (day25), 102 mm (day30), 104 mm (day40), 109 mm (day50), 104 mm (day60) of *Fgf5*^*–/–*^ mice (Figure 6B) after busulfan treatment. In Figure 6J, the total lengths of counted tubule segments were 46 mm (+DiI in FGF5 beads), 79 mm (–DiI in FGF5 beads), 17 mm (+DiI in BSA beads), 17 mm (–DiI in BSA beads). In Figure S2E, the densities of GFRα1^+^ and SOX9^+^ cells were counted in total 14- and 18-mm tubule segments of WT and *Fgf5*^*–/–*^ mice. In Figures S6M and S6N, the GFRa1^+^ or RARγ^+^ cell densities were counted in the following segments, 53 mm (day0), 32 mm (day3), 28 mm (day6), 29 mm (day9), 30 mm (day14), 39 mm (day20), 21 mm (day30). The densities of GFRα1^+^ or RARγ^+^ cells were counted in the cryo-sections at Figures 3A left, 3B–3H, 6C, S2D, S3D, S3H, S4I, S4J, S4M, S6K, and S6L were counted by the cryo-sections. For counting the cell density per tubule section, the GFRα1^+^ or RARγ^+^ cells per more than 150 tubule sections per testes (N ≥ 3 mice) were counted by Olympus BX51 microscopy.

To assess whether spermatogonia are distributed uniformly, randomly or in a clustered fashion, we performed a statistical test, comparing the homeostatic frequencies of spermatogonial unit numbers in bins of 1mm length along the tubule axis to a Poisson distribution with the same mean (Figure S1A). A standard $\chi^{2}$-test yielded a p value smaller than $10^{-5}$, indicating a significant deviation from spatial randomness. Furthermore, a variance-to-mean ratio of the bin population of $\left(\langle n^{2}\rangle -{\langle n\rangle }^{2}\right)/\langle n\rangle \approx 3$ indicated that spermatogonial units were more clustered rather than random or uniform. However, we could not detect any spatially regular patterns associated with this clustering.

#### Measurement of *Fgf5* RNA-positive cells on tubule circumference

Testicular sections were stained for *Fgf5* by *in situ* hybridization. The tubule cross sections were photographed under bright-field illumination, then measured the *Fgf5*–positive signals on tubule circumference.

#### Measurement of FGF5-positive signals adjacent to interstitial area or tubule bounding area

Testicular sections were double stained for FGF5 and CD34 by IF. The tubule cross sections were photographed under a confocal microscope (Nikon A1r). The data were then analyzed to determine whether the FGF5-positive or -negative signals distributed in the interstitial-tubule or tubule-tubule bounding regions, and then measured their lengths of FGF5- and CD34-double positive or FGF5-negative and CD34-positive, respectively.

#### Scoring the distribution of GFRα1^+^, RARγ^+^, KIT^+^ and ID4^+^ spermatogonia for *Fgf5*–positive area

Testicular sections were stained for *Gfrα1* or *Fgf5* by ISH, or RARγ, KIT, or ID4 by IHC. All the tubule cross sections were photographed. The spatial correlation with *Fgf5*–positive signals was judged from whether the distributions of *Gfrα1,* RARγ or KIT-positive spermatogonia were adjacent to *Fgf5*^+^ signals on the adjacent section. When their spermatogonia were (or were not) adjacent to *Fgf5*^+^ signals, they were categorized as “positive” (or “negative”). When their spermatogonia were partially adjacent to *Fgf5*^+^ signals, they were categorized as “boundary.” The category of “boundary” was estimated as an intermediate between “positive” and “negative,” and then the each “boundary” parameter was assigned to 0.5 of “positive” and 0.5 of “negative” in the determination whether the spermatogonia distributions were adjacent to *Fgf5* mRNA in the category between positive or negative for the calculation. Then, expected positive numbers were calculated assuming their non-biased distributions, and the ‘preferences’ (actual positive cell number/expected positive cell number) were determined; these were statistically evaluated by chi-square test between the actual and expected cell numbers adjacent or not to *Fgf5*^+^ area. For each data point, more than 40 seminiferous tubule cross-sections in 3 testicular slices were examined. The data were then analyzed to determine whether the distributions of *Gfrα1*, RARγ, KIT or ID4-positive spermatogonia were adjacent to *Fgf5*^+^ signals on the adjacent section. Statistical evaluations were performed as explained below, using the data of the *Gfrα1*-positive spermatogonia as an example. The 3 testis specimens used for analyses contained 91 cross-sections of the seminiferous tubules in total, and 121 *Gfrα1*-positive spermatogonia were observed. These 121 spermatogonia were classified as “positive” or “negative” for adjacent to *Fgf5*^+^ signals on the adjacent section. The lengths of the circumference of the tubule cross-sections were also summarized according to their levels of ISH staining of *Fgf5*. Then, the expected numbers of *Gfrα1*-positive spermatogonia in the category of positive or negative area were calculated on the basis of the null hypothesis: the *Gfrα1*-positive spermatogonia evenly distribute without bias. The expected numbers of *Gfrα1*-positive spermatogonia were obtained by multiplying the total number of *Gfrα1*-positive spermatogonia by the percentage of tubule sections in the category. The preferences of positive cells in each category were calculated as the ratio of the observed number of positive cells in each category to the expected number in the same category. Thus, a preference of 1 indicates that the actual number of *Gfrα1*-positive cells is the same as expected, i.e., a non-biased distribution. Values greater or smaller than 1 suggest preference or avoidance, respectively. The differences between the observed and expected numbers of *Gfrα1*-positive spermatogonia were statistically evaluated by chi-square tests. The *P*-value (0.00002) was small enough to reject the null hypothesis and indicated a non-random distribution of *Gfrα1*-positive spermatogonia. This was also true for RARγ but not KIT-positive spermatogonia, whose *P*-values were 0.00052 and 0.13494, respectively.

#### Scoring the distribution of GFRα1^+^ and ID4^+^ spermatogonia for the interstitium or tubule-tubule bounding area

Testicular sections were stained for GFRα1 and ID4 by IF. The tubule cross sections were photographed by Nikon A1r confocal system. The spatial correlation was judged from whether the distributions of GFRα1 or ID4-positive spermatogonia were adjacent to the interstitium or tubule bounding region. When their spermatogonia were partially adjacent to the intermediate area between the interstitium and the tubule bounding region, they were categorized as “boundary.”

### Data and Software Availability

The accession number for the microarray data reported in this study is NCBI GEO: GSE118846.

## Supporting Citations

The following references appear in the Supplemental Information: Aloisio et al., 2014, Keener and Sneyd, 2009, Strogatz, 1994.

## Supplemental Information

Document S1. Figures S1–S7

Methods S1. The ‘Mitogen Competition Model,’ Related to STAR Methods

Table S1. cDNA Clones Used for ISH Screening, Related to Figure 2

Table S2. Parameters Used for Mathematical Modeling, Related to Figures 5 and S6

Table S3. Primers Used in this Study for qRT-PCR and Genomic qPCR, Related to STAR Methods

Document S2. Article plus Supplemental Information
