# Supplementary material for: Competition for Mitogens Regulates Spermatogenic Stem Cell Homeostasis in an Open Niche
Source: Cell Stem Cell. 2019 Jan 3;24(1):79–92.e6. doi: 10.1016/j.stem.2018.11.013 (PMC6327111; doi:10.1016/j.stem.2018.11.013)
Supplement: Document S1. Figures S1–S7 [file mmc1.pdf]

Cell Stem Cell, Volume *24*

Supplemental Information

Competition for Mitogens Regulates Spermatogenic Stem Cell Homeostasis in an Open Niche

Yu Kitadate, David J. Jörg, Moe Tokue, Ayumi Maruyama, Rie Ichikawa, Soken Tsuchiya, Eri Segi-Nishida, Toshinori Nakagawa, Aya Uchida, Chiharu Kimura-Yoshida, Seiya Mizuno, Fumihiro Sugiyama, Takuya Azami, Masatsugu Ema, Chiyo Noda, Satoru Kobayashi, Isao Matsuo, Yoshiakira Kanai, Takashi Nagasawa, Yukihiko Sugimoto, Satoru Takahashi, Benjamin D. Simons, and Shosei Yoshida

Figure S1

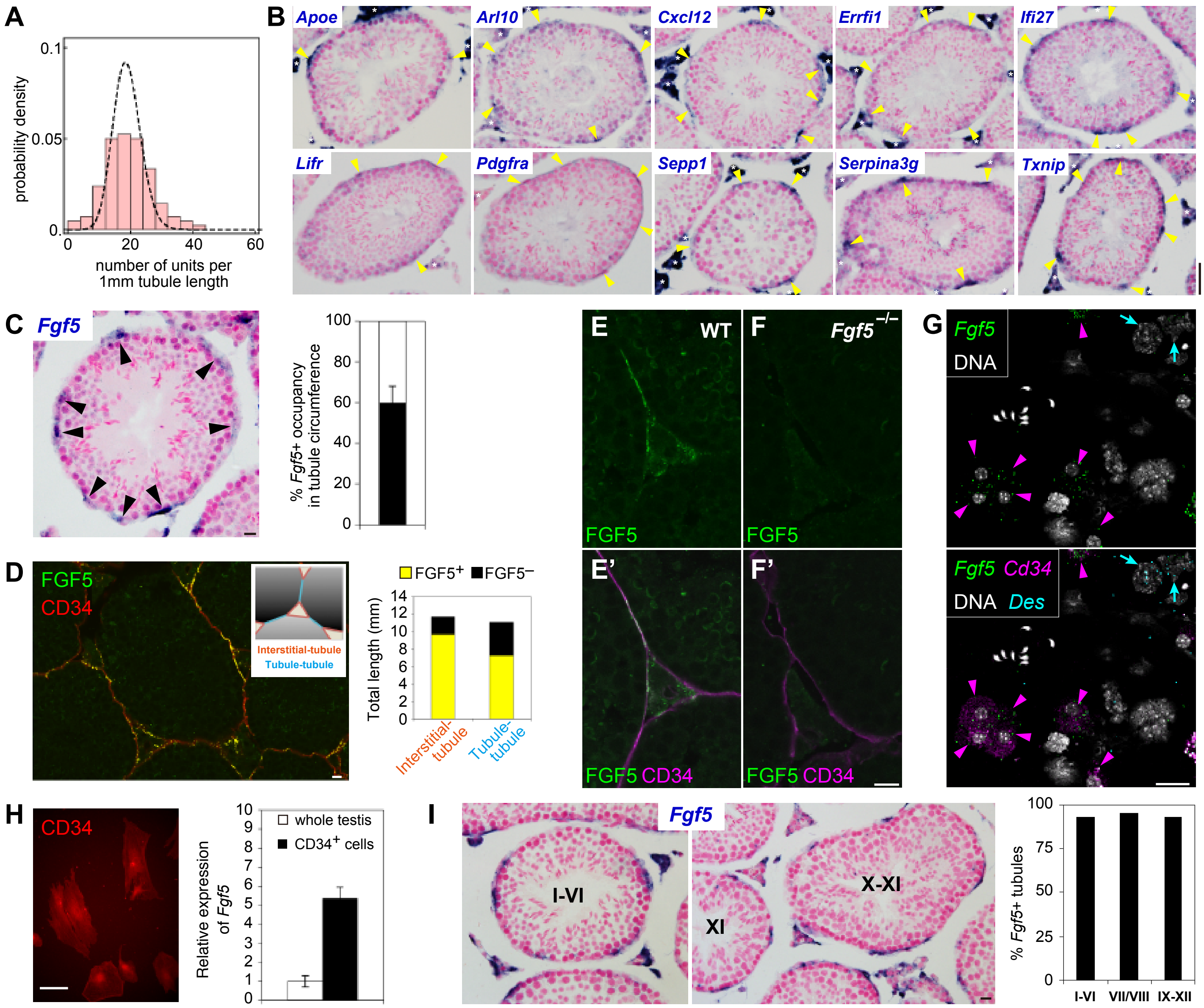

J

	Fgf5 on tubule circumference		
	positive	negative	Total
Total length of tubule circumference (mm) (a)	31.53	21.10	52.63(A)
Percentage of total length (b)	0.599	0.401	1.000

b = a/A

Observed versus expected numbers of GFRα1+ cells

Number of GFRα1+ cells	*Fgf5* on tubule circumference positive	negative	Total
Observed (c)	97.5	23.5	121(B)
Expected (d)	72.48	48.52	121
Preference (e)	1.35	0.48	

d = b x B; e = c / d; P = 0.00002, based on Chi-squared test

Observed versus expected numbers of RARγ+ cells

Number of RARγ+ cells	*Fgf5* on tubule circumference positive	negative	Total
Observed (c)	126	43	169(B)
Expected (d)	101.23	67.77	169
Preference (e)	1.24	0.63	

d = b x B; e = c / d; P = 0.00052, based on Chi-squared test

Observed versus expected numbers of KIT+ cells

Number of KIT+ cells	*Fgf5* on tubule circumference positive	negative	Total
Observed (c)	233	125	358(B)
Expected (d)	214.44	143.56	358
Preference (e)	1.09	0.87	

d = b x B; e = c / d; P = 0.13494, based on Chi-squared test

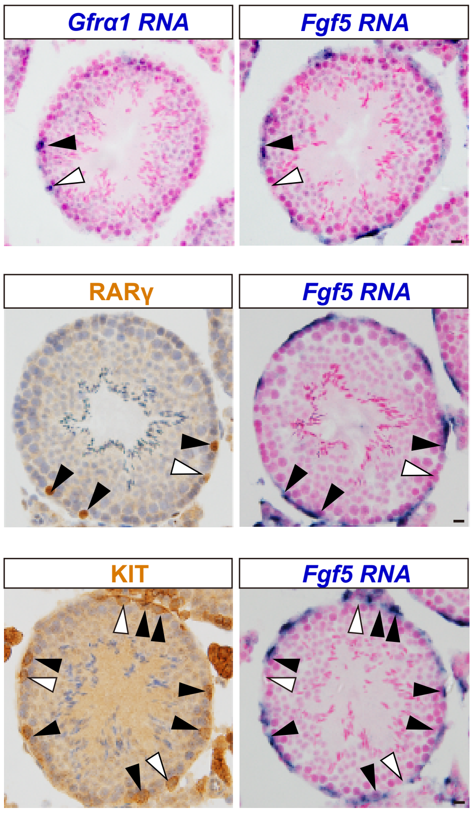

Figure S1. Extended analyses of FGF5 expression, Related to Figure 2.

(A) Experimentally determined number distribution of GFRα1$^+$ spermatogonial units (i.e., mononucleated [A_s] cells or syncytia [A_{pr} or A_{al}]; Hara et al., 2014) per 1 mm tubule length (bars) compared with a Poisson distribution with the same mean that would correspond to complete spatial randomness. (B) Expression patterns of the indicated transcripts within subset peritubular flattened cells near the interstitium and intertubular arterioles/venules (asterisks), revealed by ISH (blue, arrowheads) with counterstaining using nuclear fast red. Scale bar, 50 μm. (C) *Left:* A representative ISH image of a cross section of seminiferous tubule stained for *Fgf5* (arrowheads). *Right:* Portion of the *Fgf5*$^+$ region in the tubule circumference, shown in average ± SEM from 3 mice (82 tubule sections in total). (D) *Left*: A representative IF image of a testis section stained for FGF5 (green) and CD34 (red), computationally modified to display only the CD34$^+$ peripheral region of the tubules with contrasted FGF5 signal using a Nikon A1r confocal system. *Right*: Lengths of FGF5-positive and negative regions of the tubule circumference, showing interstitial-tubule or tubule-tubule contact (inset). Measurements of 31 tubule sections are summarized. (E–F) IF images for FGF5 (green) and CD34$^+$ (red) in WT (E and E') and *Fgf5*$^{–/–}$ (F and F') testis sections, verifying the specificity of FGF5 staining. (G) Multicolor fluorescent RNA ISH on dissociated testicular cells, showing the expression of *Fgf5* (green) in some *Cd34*$^+$ (magenta) LE, but not in *Desmin* (*Des*)$^+$ (cyan) myoid, cells. (H) *Left*: Cultured mouse testicular cells (see STAR Methods), immunostained for CD34. *Right*: RT-qPCR analysis for *Fgf5* expression in these cells, compared with the whole testis (Average ± SEM, N=4). (I) *Left*: Representative ISH images of testis sections for *Fgf5* in tubule segments at different stages of seminiferous epithelial cycle (indicated by Roman numerals). *Right*: Percent tubule sections at the indicated stages that accompany one or more *Fgf5*$^+$ cells. N≥10 tubule sections of the stages. (J) Distribution of GFRα1$^+$, RARγ$^+$ or KIT$^+$ spermatogonia relative to *Fgf5*$^+$ LE cells. *Left*: Quantification of their positional relationship. *Right*: Examples of adjacent section pairs stained for *Fgf5* and *Gfrα1*, RARγ or KIT. Filled and open arrowheads show *Gfrα1*$^+$, RARγ$^+$ or KIT$^+$ spermatogonia located in the *Fgf5*-positive and negative areas, respectively. Scale bars, 10 μm in (C–J).

Figure S2

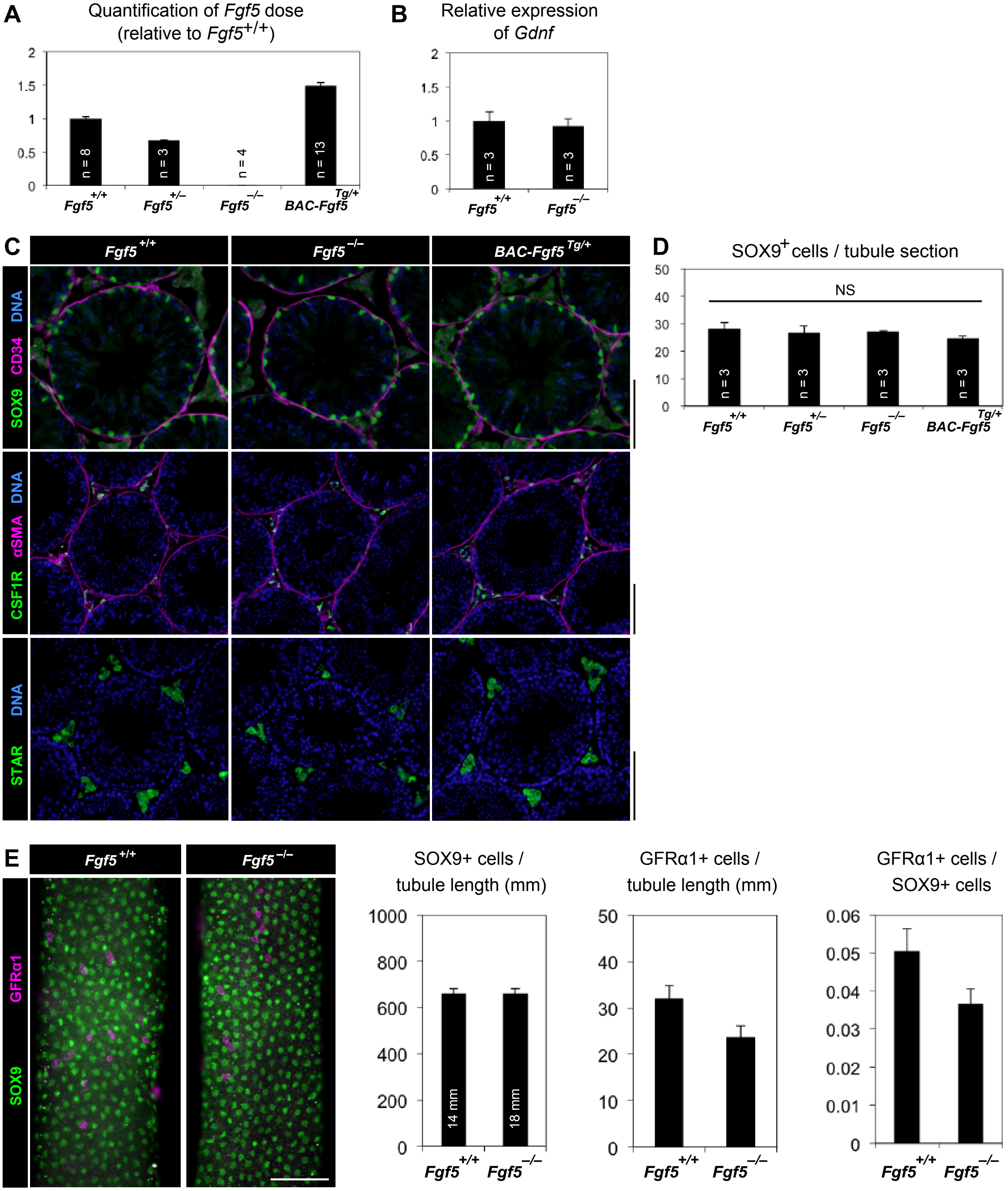
A
Quantification of Fgf5 dose
(relative to Fgf5+/+)
2
1.5
1
0.5
0
n = 8
n = 3
n = 4
n = 13
Fgf5+/+
Fgf5+/–
Fgf5–/–
BAC-Fgf5Tg/+
B
Relative expression
of Gdnf
2
1.5
1
0.5
0
n = 3
n = 3
Fgf5+/+
Fgf5–/–
C
Fgf5+/+
Fgf5–/–
BAC-Fgf5Tg/+
DNA
CD34
SOX9
DNA
αSMA
CSF1R
DNA
STAR
D
SOX9+ cells / tubule section
50
40
30
20
10
0
NS
n = 3
n = 3
n = 3
n = 3
Fgf5+/+
Fgf5+/–
Fgf5–/–
BAC-Fgf5Tg/+
E
Fgf5+/+
Fgf5–/–
GFRα1
SOX9
SOX9+ cells /
tubule length (mm)
1000
800
600
400
200
0
14 mm
18 mm
Fgf5+/+
Fgf5–/–
GFRα1+ cells /
tubule length (mm)
50
40
30
20
10
0
Fgf5+/+
Fgf5–/–
GFRα1+ cells /
SOX9+ cells
0.06
0.05
0.04
0.03
0.02
0.01
0
Fgf5+/+
Fgf5–/–

Figure S2. Supplemental characterization of *Fgf5* mutant mice, Related to Figure 3.
(A) Genomic qPCR quantification of the dosage of *Fgf5* gene, indicating one copy per *BAC-Fgf5 Tg* allele, shown in average ± SEM. n = number of animals examined. (B) RT-qPCR analysis of *Gdnf* expression in WT and *Fgf5*$^{-/-}$ mutant mouse testis. Average ± SEM values from 3 animals for each genotype are shown. (C) Representative IF images of testicular sections for SOX9, CD34, CSF1R, αSMA and STAR (markers of Sertoli, lymphatic endothelial, macrophagic, myoid and Leydig cells, respectively). (D) Number of SOX9^{+} Sertoli cells per tubule section in *Fgf5* mutants. N> 200 tubule sections from 3 mice were counted for each genotype, shown in average ± SEM. (E) *Left*: Representative IF images of whole mount seminiferous tubules stained for SOX9 (green) and GFRα1 (magenta). *Right*: Number of SOX9^{+} Sertoli cells and GFRα1^{+} spermatogonia per mm tubule length and their ratio. Totally 14 (WT) and 18 (*Fgf5*$^{-/-}$) mm-long tubule segments from 3 mice were counted.

Throughout, average values ± SEM are shown. Scale bars, 100 μm. **$p<0.05$ versus *Fgf5*$^{+/+}$; NS, not significant (t-test).

Figure S3

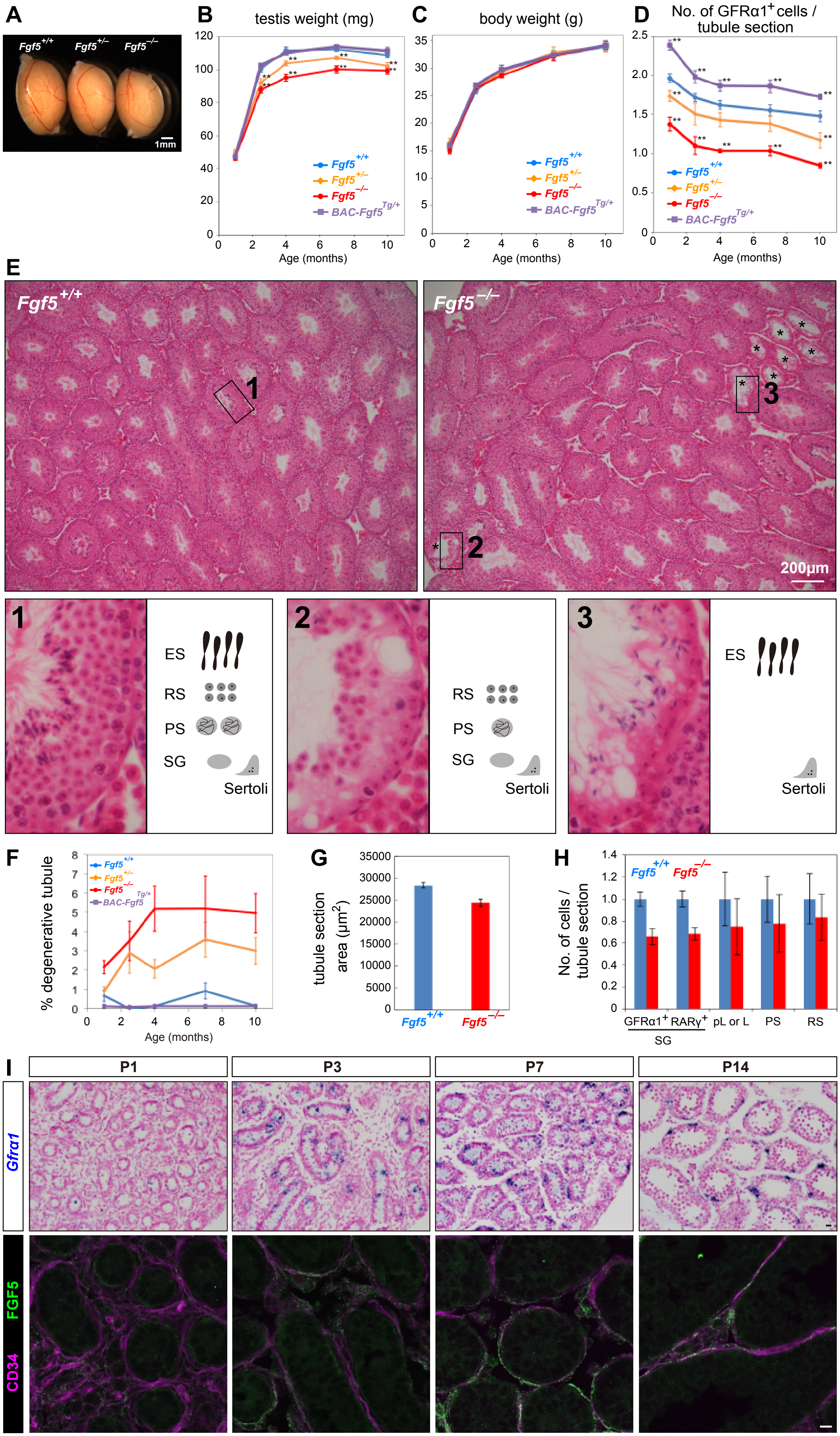

A
Fgf5+/+ Fgf5+/– Fgf5–/–
1mm
B
testis weight (mg)
Age (months)
Fgf5+/+
Fgf5+/–
Fgf5–/–
BAC-Fgf5Tg/+
C
body weight (g)
Age (months)
D
No. of GFRα1+ cells / tubule section
Age (months)
E
Fgf5+/+
Fgf5–/–
200μm
ES
RS
PS
SG
Sertoli
F
% degenerative tubule
Age (months)
G
tubule section area (μm2)
H
No. of cells / tubule section
GFRα1+ RARγ+ pL or L PS RS
SG
I
P1 P3 P7 P14
Gfra1
FGF5
CD34

Figure S3. Supplemental characterization of spermatogenesis in *Fgf5* mutant mice and FGF5 expression during postnatal development, Related to Figure 3.

(A) Appearance of the testes of *Fgf5*$^{+/+}$, *Fgf5*$^{+/–}$ and *Fgf5*$^{–/–}$ mice at 2.5 months of age. (B–C) Testis (B) and body (C) weights of *Fgf5* mutants at the indicated ages. (D) Absolute density of GFRα1^{+} cells in seminiferous tubules of *Fgf5* mutants at the indicated ages. (E) H&E-stained sections of *Fgf5*$^{+/+}$ and *Fgf5*$^{–/–}$ testes. Lower panels show the examples of normal (1) and affected (2, 3) tubules indicated, with schemes representing the residual cell types. Asterisks indicate degenerative tubules. (F) Proportions of seminiferous tubules harboring degenerated spermatogenesis in *Fgf5* mutants at the indicated ages. (G) Area of transverse section of seminiferous tubules of WT and *Fgf5*$^{–/–}$ mice at 4 months of age, measured in sections that appear in round shapes. (H) Relative numbers of indicated spermatogenic cells counted in tubule sections in *Fgf5*$^{+/+}$ and *Fgf5*$^{–/–}$ 2.5-month-old mice. (I) Representative images of ISH for *Gfrα1* and IF for FGF5 (green) and CD34 (magenta) on postnatal testis sections at the indicated ages.

Scale bars indicate 200 and 10 μm in (E) and (I), respectively. Average values ± SEM from N≥3 mice for each data point in (B–D, F–H). SG, spermatogonia; pL, preleptotene spermatocytes; L, leptotene spermatocytes; PS, pachytene spermatocytes; RS, round spermatids; ES, elongated spermatids. pL or L, PS and RS were counted in stages VII and VIII (H).

Figure S4

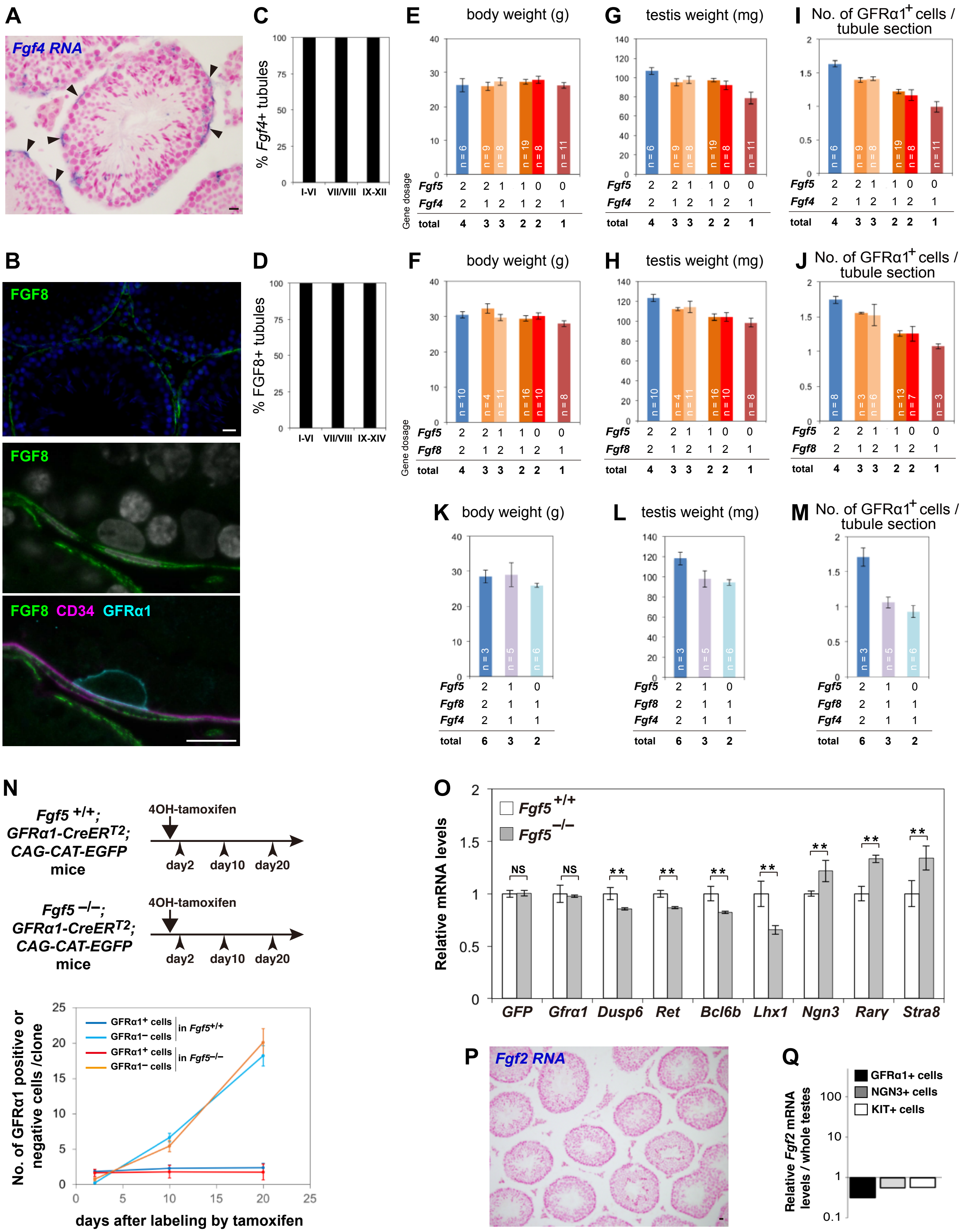

A
Fgf4 RNA
B
FGF8
FGF8
FGF8 CD34 GFRα1
C
% Fgf4+ tubules
I-VI VII/VIII IX-XII
D
% FGF8+ tubules
I-VI VII/VIII IX-XIV
E body weight (g)
G testis weight (mg)
I No. of GFRα1+ cells / tubule section
F body weight (g)
H testis weight (mg)
J No. of GFRα1+ cells / tubule section
Gene dosage
Fgf5 2 2 1 1 0 0
Fgf4 2 1 2 1 2 1
Fgf8 2 1 2 1 2 1
total 4 3 3 2 2 1
K body weight (g)
L testis weight (mg)
M No. of GFRα1+ cells / tubule section
Fgf5 2 1 0
Fgf8 2 1 1
Fgf4 2 1 1
total 6 3 2
N
Fgf5 +/+; GFRα1-CreERT2; CAG-CAT-EGFP mice
Fgf5 −/−; GFRα1-CreERT2; CAG-CAT-EGFP mice
4OH-tamoxifen
day2 day10 day20
No. of GFRα1 positive or negative cells /clone
days after labeling by tamoxifen
GFRα1+ cells
GFRα1− cells
in Fgf5+/+
in Fgf5−/−
O
Fgf5+/+
Fgf5−/−
Relative mRNA levels
NS NS ** ** ** ** ** ** **
GFP Gfrα1 Dusp6 Ret Bcl6b Lhx1 Ngn3 Rarγ Stra8
P
Fgf2 RNA
Q
GFRα1+ cells
NGN3+ cells
KIT+ cells
Relative Fgf2 mRNA levels / whole testes

Figure S4. Expression of *Fgf4* and FGF8, and supplemental testicular phenotypes of *Fgf4* and *Fgf8* mutant mice, Related to Figure 3.

(A) A representative ISH image for *Fgf4* on a testis section, showing its expression in flattened peritubular cells (arrowheads), counter-stained with nuclear fast red. (B) Representative IF images of rat testis section stained for FGF8 (green), CD34 (magenta) and GFRα1 (cyan). (C–D) Percent tubule sections at the indicated stages that accompany one or more *Fgf4*$^{+}$ (C) or FGF8^{+} (D) cells, respectively, indicating their expression throughout the cycle. N≥10 tubule sections for each group of the stages. (E–M) Body weights (E, F, K), testis weights (G, H, L) and the absolute densities of GFRα1^{+} cells (I, J, M) in mice harboring the indicated dosages of functional *Fgf5*, *Fgf8*, and *Fgf4* alleles at 2.5 months of age. Averages ± SEM are shown; n = number of animals examined. (N) Clonal fates of pulse-labeled GFRα1^{+} cells in *Fgf5*$^{+/+}$ and *Fgf5*$^{-/-}$ mice, from experiments conducted following the schedule on the top. Average numbers ± SEM of GFRα1^{+} and GFRα1^{-} cells contained in a single clone are shown. ≥187 clones in ≥4 testes for each data point were analyzed. (O) RT-qPCR analysis of the expression levels of indicated genes in GFRα1-GFP^{+} cells sorted from testicular cells of *Fgf5*$^{+/+}$ and *Fgf5*$^{-/-}$ mice that also harbor a *Gfrα1-gfp* knock-in allele. Average values ± SEM from 3 independent preparations are shown. (P) A representative ISH image of a testis section for *Fgf2*, showing no significant staining. (Q) Expression of *Fgf2* in GFRα1^{+}, NGN3^{+} and KIT^{+} spermatogonia, selected from published cDNA microarray data (Ikami et al., 2015) and normalized to the values from the whole testis. Scale bars, 10μm in (A–B) and (P).

Figure S5

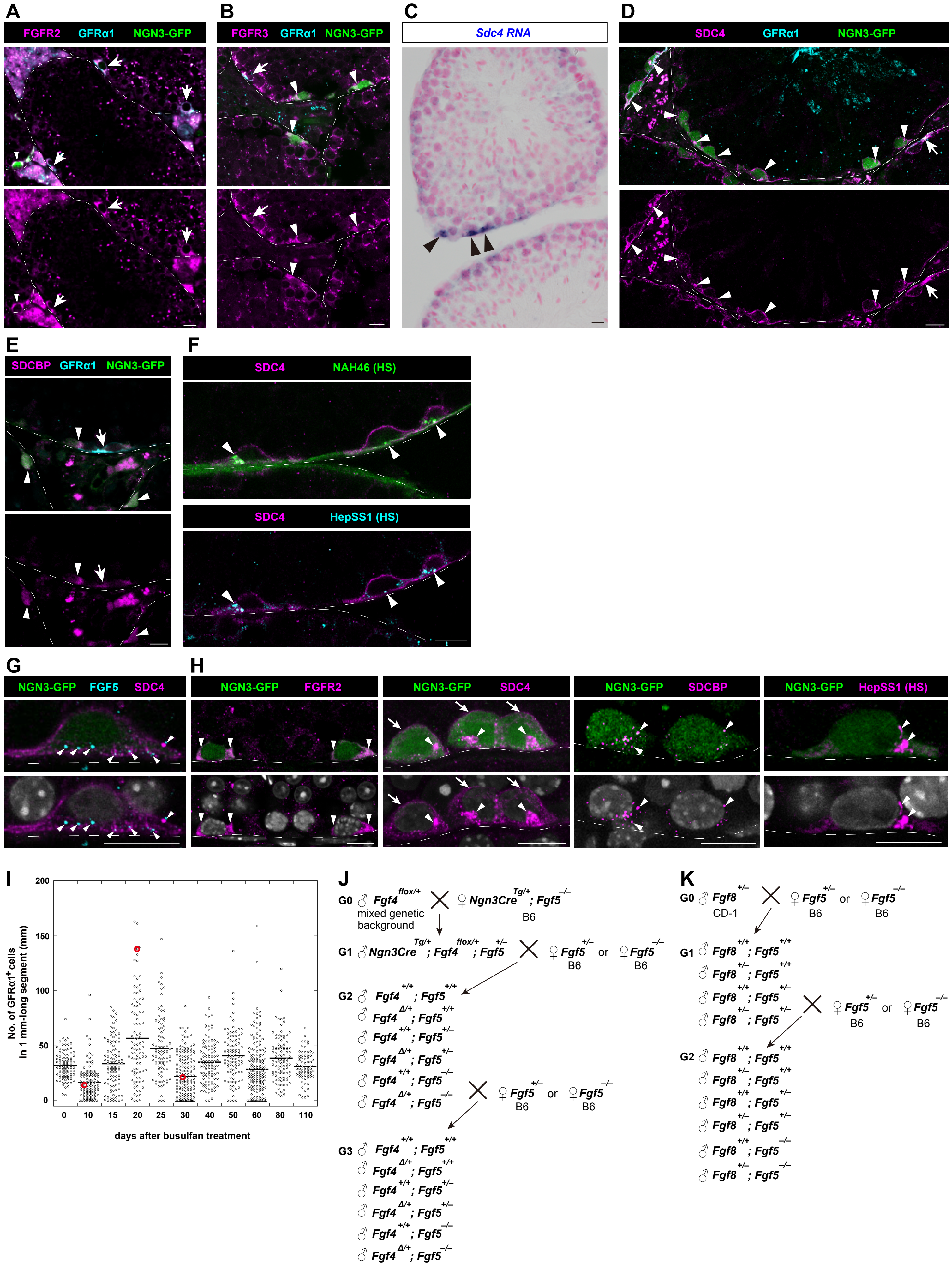

A
FGFR2 GFRα1 NGN3-GFP
B
FGFR3 GFRα1 NGN3-GFP
C
Sdc4 RNA
D
SDC4 GFRα1 NGN3-GFP
E
SDCBP GFRα1 NGN3-GFP
F
SDC4 NAH46 (HS)
SDC4 HepSS1 (HS)
G
NGN3-GFP FGF5 SDC4
H
NGN3-GFP FGFR2
NGN3-GFP SDC4
NGN3-GFP SDCBP
NGN3-GFP HepSS1 (HS)
I
No. of GFRα1+ cells in 1 mm-long segment (mm)
200
150
100
50
0
0 10 15 20 25 30 40 50 60 80 110
days after busulfan treatment
J
G0 ♂ Fgf4 flox/+ × ♀ Ngn3Cre Tg/+ ; Fgf5 –/–
mixed genetic background
B6
G1 ♂ Ngn3Cre Tg/+ ; Fgf4 flox/+ ; Fgf5 +/– × ♀ Fgf5 +/– or ♀ Fgf5 –/–
B6 B6
G2 ♂ Fgf4 +/+ ; Fgf5 +/+
♂ Fgf4 Δ/+ ; Fgf5 +/+
♂ Fgf4 +/+ ; Fgf5 +/–
♂ Fgf4 Δ/+ ; Fgf5 +/–
♂ Fgf4 +/+ ; Fgf5 –/–
♂ Fgf4 Δ/+ ; Fgf5 –/–
× ♀ Fgf5 +/– or ♀ Fgf5 –/–
B6 B6
G3 ♂ Fgf4 +/+ ; Fgf5 +/+
♂ Fgf4 Δ/+ ; Fgf5 +/+
♂ Fgf4 +/+ ; Fgf5 +/–
♂ Fgf4 Δ/+ ; Fgf5 +/–
♂ Fgf4 +/+ ; Fgf5 –/–
♂ Fgf4 Δ/+ ; Fgf5 –/–
K
G0 ♂ Fgf8 +/– × ♀ Fgf5 +/– or ♀ Fgf5 –/–
CD-1 B6 B6
G1 ♂ Fgf8 +/+ ; Fgf5 +/+
♂ Fgf8 +/– ; Fgf5 +/+
♂ Fgf8 +/+ ; Fgf5 +/–
♂ Fgf8 +/– ; Fgf5 +/–
× ♀ Fgf5 +/– or ♀ Fgf5 –/–
B6 B6
G2 ♂ Fgf8 +/+ ; Fgf5 +/+
♂ Fgf8 +/– ; Fgf5 +/+
♂ Fgf8 +/+ ; Fgf5 +/–
♂ Fgf8 +/– ; Fgf5 +/–
♂ Fgf8 +/+ ; Fgf5 –/–
♂ Fgf8 +/– ; Fgf5 –/–

Figure S5. FGF uptake by NGN3$^+$ spermatogonia, Related to Figure 4.

(A–B) Representative IF images of NGN3-GFP mouse testis stained for GFP (green), GFRα1 (cyan), and FGFR2 (A) or FGFR3 (B) (magenta). FGFR2 and FGFR3 were stained in GFRα1$^+$ (arrows) and NGN3-GFP$^+$ cells (arrowheads). (C) A representative ISH image stained for *Sdc4* (blue) in a testis section, enriched in a few spermatogonia (arrowheads). (D–E) Representative IF images of NGN3-GFP mouse testis stained for GFP (green), GFRα1 (cyan), and SDC4 (D) or SDCBP (E) (magenta). SDC4 and SDCBP were stained in GFRα1$^+$ (arrows) and NGN3-GFP$^+$ cells (arrowheads). (F) A representative IF image of testis sections, stained with NAH46 (recognizing all HS, green), HepSS1 (recognizing highly sulfated HS, cyan), and anti-SDC4 (magenta) antibodies. NAH46 immunoreactivity was accumulated both in the basement membrane and the cytoplasm of SDC4$^+$ cells (arrowheads). In contrast, that of HepSS1 was specifically enriched in the cytoplasm of SDC4$^+$ cells (arrowheads). (G–H) Representative IF images of NGN3-GFP$^+$ (green) cells exhibiting punctate staining for SDC4, FGFR2, SDCBP or HS (stained with HepSS1) (magenta), or FGF5 (cyan). Arrowheads indicate their cytoplasmic staining, with arrows indicating cell surface SDC4 staining. (I) Quantification of GFRα1$^+$ cell density after perturbation from homeostasis. According to the schedule shown in Figure 6A, the number of GFRα1$^+$ cells contained in seminiferous tubules of wild-type testes were counted using whole-mount immunofluorescence. Each dot shows the number of GFRα1$^+$ cells in each 1 mm-long tubule segment, and the horizontal bars indicate the average values. Average ± SEM (N ≥4 for each time point) from these data are indicated in Figure 6A (left). IF images corresponding to the data point indicated by red circles are shown in Figure 6A (right panels). (J–K) Mating scheme to obtain mice harboring *Fgf5* and *Fgf4* (J) and *Fgf5* and *Fgf8* (K) mutations. *Fgf4*$^{flox/+}$ and *Fgf8*$^{+/–}$ males with mixed and CD-1 background, respectively, and their offspring were crossed with females with C57BL/6J (B6) background carrying *Fgf5*$^{–}$ allele and/or *Ngn3-Cre* transgene (used to recombine the *Fgf4*^{flox} allele to generate the null, *Fgf4*$^{\Delta}$ allele), as indicated.

In (A–H), broken lines show the periphery of seminiferous tubules; scale bars indicate 10 μm.

Figure S6

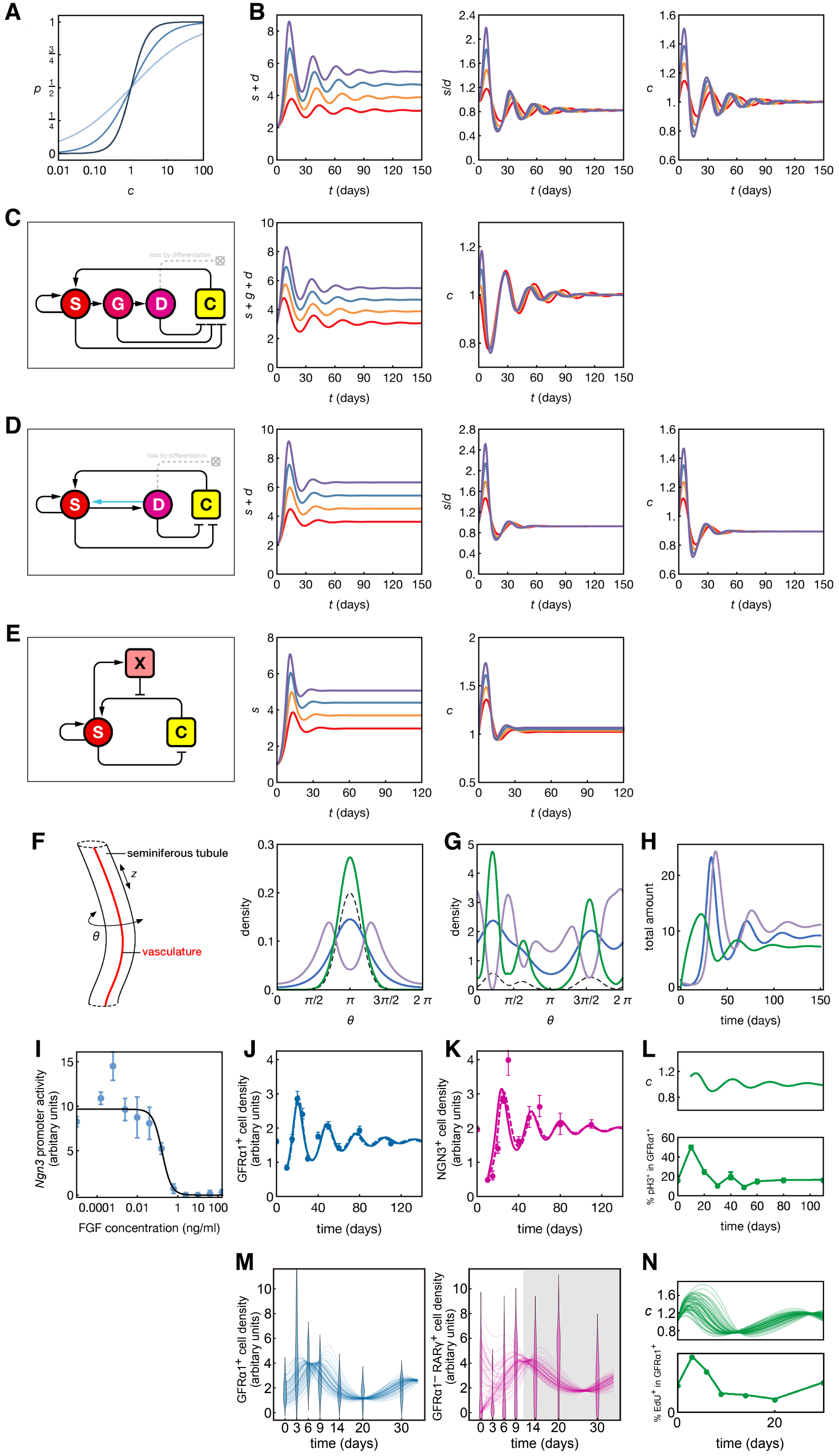

A
B
C
D
E
F
G
H
I
J
K
L
M
N
p
c
s + d
s/d
t (days)
loss by differentiation
S
G
D
C
X
s + g + d
s
seminiferous tubule
z
θ
vasculature
density
total amount
time (days)
Ngn3 promoter activity (arbitary units)
FGF concentration (ng/ml)
GFRα1⁺ cell density (arbitary units)
NGN3⁺ cell density (arbitary units)
% pH3⁺ in GFRα1⁺
GFRα1− RARγ⁺ cell density (arbitary units)
% EdU⁺ in GFRα1⁺

Figure S6. Mitogen competition model, Related to Figure 5, Figure 6, STAR Methods, and Methods S1

(A) Examples for the function $p(c)$, given by Eq. (3), that mediates the feedback of the mitogen concentration on the spermatogonial fates, for $c_0 = 1$ and $m = 1/2$ (light blue), $m = 1$ (mid-blue), and $m = 2$ (dark blue). (B) Numerical examples of the model with two stem cell compartments, Eqs. (8), as schematically shown in Figure 5A'. *Left:* Total progenitor cell density $s + d$ as a function of time. *Center:* Ratio of cell densities s/d as a function of time. *Right:* Mitogen concentration c as a function of time. Different curves show different mitogen production rates: $\mu/c_0 = 0.22d^{-1}$(red), $\mu/c_0 = 0.26d^{-1}$ (orange), $\mu/c_0 = 0.30d^{-1}$ (blue), $\mu/c_0 = 0.34d^{-1}$ (purple). The other parameters are given in Supplemental Table 2. Initial conditions are $s(0) = d(0) = 1$ (in units of the reference cell density) and $c(0) = 1$ (in units of c_0). (C) Variant of the mitogen competition model with self-renewal by only a subcompartment of cells, Eqs. (10). *Left:* Model scheme. *Center:* Total progenitor cell density $s + g + d$ as a function of time. *Right:* Mitogen concentration c as a function of time. All parameters and conventions as in panel (B); additional parameters are $\lambda' = \lambda$ and $\varepsilon = 0.1$. Initial conditions are $s(0) = g(0) = d(0) = 1$ (in units of the reference cell density) and $c(0) = 1$ (in units of c_0). (D) Variant of the mitogen competition model that includes reversion of differentiating cells to the stem cell compartment, Eqs. (11). *Left:* Model scheme. All other displayed quantities, parameters and conventions as in panel (B); the reversion rate is $\bar{\gamma} = \gamma/4$. (E) Model variant including an antagonizing factor, Eqs. (15). *Left:* Model scheme. *Center:* Stem cell density s as a function of time. *Right:* Mitogen concentration c as a function of time. All parameters and conventions as in panel (B); additional parameters are $\alpha\varrho/x_0 = 0.05d^{-1}$ and $\beta = 1d^{-1}$ with ϱ being the unit of cell density. (F–H) Spatially extended model including stem cell motion, Eqs. (16). For all panels, parameters are given in Supplemental Table 2 with $\tilde{\eta} = 0.25$. (F) *Left:* Sketch of the model geometry. *Right:* Steady state density distributions for stem cells ϕ^S (blue), progenitors ϕ^D (pink) and mitogens ϕ^C (green) with a single mitogen source with angular extension $\sigma = 0.5$ located at π (dashed curve). (The mitogen concentration has been scaled by a factor of 0.1.) (G) The same as in panel (F) but with 5

randomly distributed mitogen sources of angular extension $\sigma = 0.35$. The dashed curve shows the source distribution J . (Mitogen concentration not rescaled.) (H) Total cell and mitogen densities as a function of time, as given by Eqs. (19), for the source distribution shown in panel (G). (I) *Ngn3* promoter activity as a function of the FGF concentration as measured in an *in vitro* luciferase assay. The dots show experimental data points, the curve is a fit of the function $r_0(1 - p(c))$ to the experimental data. The fit parameters and corresponding standard errors are c_0 = 0.17 ± 0.04 ng/ml, m = 1.9 ± 0.8 and r_0 = 9.7 ± 0.6 ng/ml. (J–K) Comparison of models with and without mitogen consumption by stem and differentiating cells to experimental data (also shown in Figure 6B and 6C). In both plots, solid lines show the best fit if only s consumes the mitogen, dashed lines show the best fit if both s and d consume the mitogen. (J) GFRα1$^+$ cell density (dots) compared to the stem cell density s in the model. (K) NGN3$^+$ cell density (dots) compared to the cell density *d* of the differentiating compartment in the model. (L) Comparison of the model prediction for the mitogen concentration c during the recovery phase in wildtype (top) and the experimentally measured percentage of pH3$^+$ among the GFRα1$^+$ cells (bottom) during the recovery phase after busulfan treatment. (M) Comparison of model predictions on the cell density kinetics after transplantation of FGF5-soaked beads. Distribution plots show the distribution of measured cell densities of different beads for GFRα1$^+$ cells (blue) and RARγ$^+$ cells (pink). Curves show model results using the experimental initial conditions while using the initial mitogen concentration $c(t)|_{t=0}$ as a fit parameter. The shaded area shows regions where the model fails to accurately describe the behavior of differentiating cells, which is likely due to seminiferous cycle-related effect not captured by our model. (N) Model predictions for the FGF concentration (curves) corresponding to the simulations shown in panel M and experimental data on the fraction of EdU$^+$ cells among the GFRα1$^+$ population after bead transplantation.

Figure S7

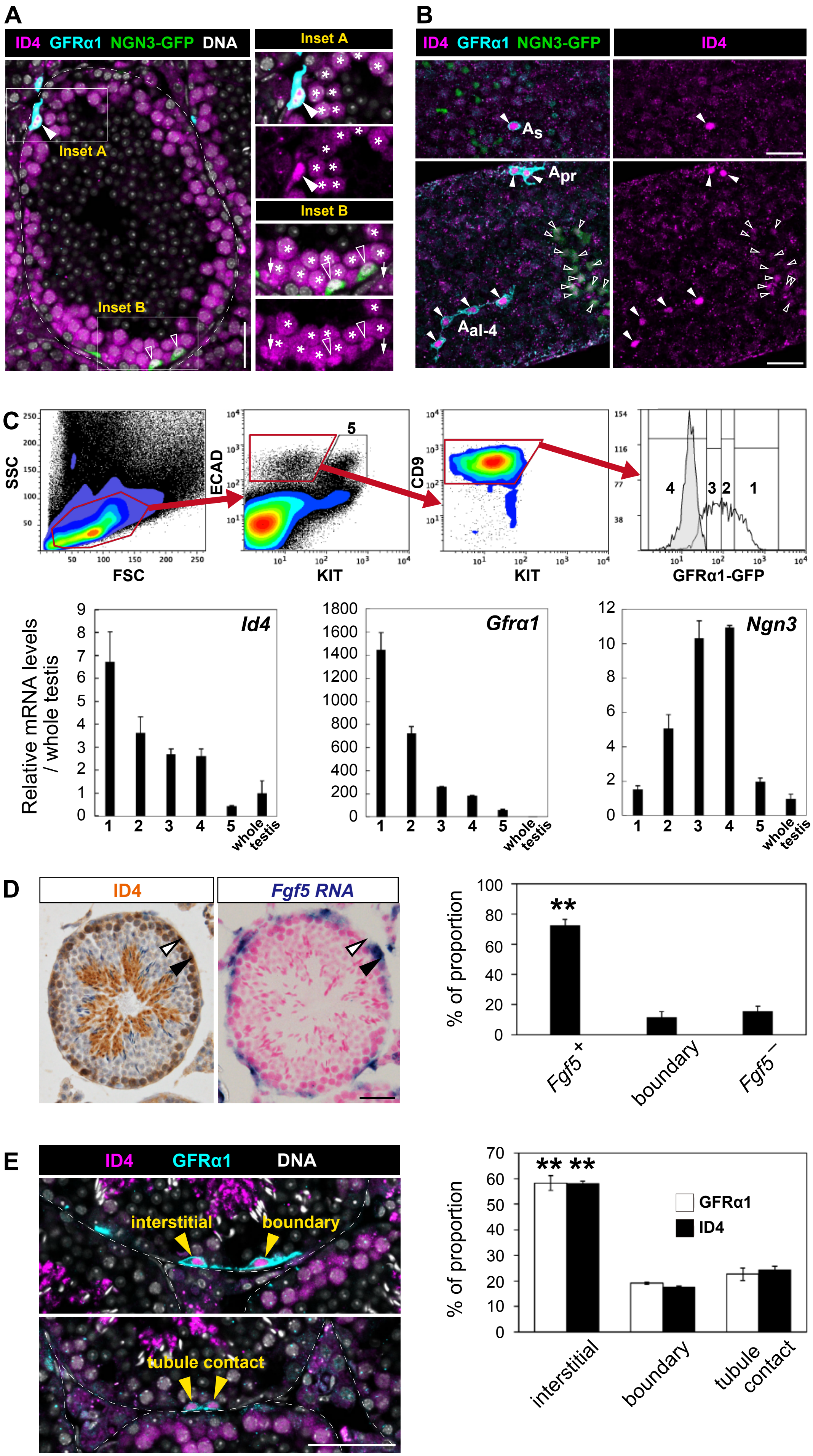

A
ID4 GFRα1 NGN3-GFP DNA
Inset A
Inset B
B
ID4 GFRα1 NGN3-GFP
ID4
As
Apr
Aal-4
C
SSC
FSC
ECAD
KIT
5
CD9
GFRα1-GFP
4
3
2
1
Relative mRNA levels / whole testis
Id4
Gfrα1
Ngn3
whole testis
D
ID4
Fgf5 RNA
% of proportion
**
Fgf5+
boundary
Fgf5−
E
ID4 GFRα1 DNA
interstitial
boundary
tubule contact
GFRα1
ID4
** **
interstitial
boundary
tubule contact

Figure S7. Analysis of ID4 expression, Related to Figure 2.

(A, B) Representative IF images of a transverse section (A) and a part of a whole-mount specimen (B) of seminiferous tubules of NGN3-GFP mice, stained for GFP (green), ID4 (magenta) and GFRα1 (cyan). Insets show the magnified images of the indicated areas. Filled and open arrowheads indicate the ID4 expression in GFRα1^{+} and NGN3-GFP^{+} spermatogonia, respectively, in addition to the broad expression in pachytene spermatocytes (asterisks) and Sertoli cells (arrows). ID4 immunoreactivity was detected in GFRα1^{+} A_{s}, A_{pr}, and $A_{al\text{-}4}$ spermatogonia, as well as in longer chains of NGN3-GFP^{+} cells to a weaker extent. (C) *Upper*: Representative flow cytometry profiles upon fractionation of cells contained in the indicated gated regions (1–5) from adult GFRα1-GFP mouse testis. The grey histogram in the right-most panel indicates the wild-type control. *Lower*: RT-qPCR analyses of the expression of indicated genes in fractions 1-5, shown in average ± SEM (n=3 mice). (D) Distribution of ID4^{+} cells relative to *Fgf5*$^{+}$ cells. *Left*: A representative pair of adjacent testicular sections stained for ID4 by IHC and *Fgf5* RNA by ISH. Filled and open arrowheads show the position of ID4^{+} spermatogonia adjacent to *Fgf5*-positive and negative areas, respectively. *Right*: Quantification of the localization of ID4^{+} spermatogonia relative to *Fgf5*$^{+}$ cells, shown in average ± SEM (n=6 mice, total of 168 cells). **P<0.05 compared to *Fgf5*-negative area (t-test). (E) *Left*: Representative examples of ID4^{+} spermatogonia showing the indicated localizations, observed on IF sections stained in the same manner with (A–B). *Right*: Quantification of the localization of ID4^{+} spermatogonia relative to interstitium, shown by average ± SEM (n=3 mice, >100 cells per mouse). **P<0.05 compared to tubule-tubule localization (t-test). Scale bars, 50 µm in (A–B) and (D–E) .

Together, Id4 is widely expressed across, or even beyond, the GFRα1^{+} population, consistent with La et al., 2018, and that Id4$^{+/high}$ cells are spatially correlated with FGF5^{+} LE cells and interstitium (and accompanying arterioles/venues). These may appear inconsistent with Chan et al., 2014, likely reflecting the different ways of detection (endogeneous protein vs *Id4-gfp* transgene), ages (adult vs various [mainly juvenile] ages), and the criteria of vasculature-associated regions (arterioles/venues accompanying interstitium vs thicker vessels without interstitium). We refer to Yoshida et al, 2018a for details.
