# Supplementary material for: Competition for Mitogens Regulates Spermatogenic Stem Cell Homeostasis in an Open Niche
Source: Cell Stem Cell. 2019 Jan 3;24(1):79–92.e6. doi: 10.1016/j.stem.2018.11.013 (PMC6327111; doi:10.1016/j.stem.2018.11.013)
Supplement: Table S3. Primers Used in this Study for qRT-PCR and Genomic qPCR, Related to STAR Methods [file mmc5.pdf]

Methods S1: The 'Mitogen Competition Model', related to STAR Methods

Model with a single stem cell compartment

The model introduced here describes the dynamics of the average density s of self-renewing stem cells and the average concentration c of mitogens within the seminiferous tubules. Here, c includes the sum of concentrations of all mitogens that affect differentiation and proliferation of stem cells in a similar manner (including *Fgf5*, *Fgf8* and *Fgf4* in this case). (Given the uncertainty in the range of the stem cell compartment, we later address the scenario in which only a subpopulation of the mitogen-consuming cells is able to self-renew.)

In our model, mitogens are produced at rate μ, decay at a rate k, and are consumed by stem cells at a rate $2k'p(c)$, where $2k'$ is the maximum consumption rate. The function $p(c)$ parameterizes the dependence of the consumption rate on the mitogen concentration c. To account for the saturation of mitogen receptor binding for large mitogen concentrations, we require $p(c)$ to satisfy the condition $p(0) = 0$ and to approach 1 for large c. Stem cells duplicate at a rate r_+ and differentiate at a rate r_-, so that the total net gain or loss rate is given by $R = r_+ - r_-$. Homeostasis corresponds to the state in which both these rates are at balance, $r_+ = r_-$, so that the cell density does not change on average. In our model, one or both of these rates may depend on the mitogen concentration c; for the competition mechanism to ensure homeostasis, it only matters that for a specific mitogen concentration c_0, the total net rate vanishes, $R(c_0) = 0$, and that certain stability conditions are met, as explained below. For concreteness, we here consider the stem cells to choose between duplication with probability q or differentiation with probability $1 - q$ at a rate λ. Hence, duplication leads to an increasing number of self-renewing cells at a rate $r_+ = q\lambda$ while differentiation leads to a loss of stem cells at a rate $r_- = (1 - q)\lambda$, so that the total net rate is given by $R = (2q - 1)\lambda$. The probability q depends on the mitogen concentration c such that cells tend to duplicate if they reveice high doses of mitogen and tend to differentiate if they receive low doses of mitogen. Since we must assume that the dependence of q on the mitogen concentration c is likewise affected by saturation of mitogen receptor binding, we here choose $q = p(c)$ for simplicity. Hence, the dynamic equations of our model are given by

$$\dot{s} = (2p(c) - 1)\lambda s \tag{1}$$

$$\dot{c} = \mu - kc - 2k'p(c)s \tag{2}$$

where the dot denotes the time derivative. A simple choice for p satisfying the above requirements is the Hill-type function (Keener 2009),

$$p(c) = \frac{(c/c_0)^m}{1 + (c/c_0)^m}, \tag{3}$$

where the exponent m determines how sensitively p depends on c, and c_0 is the reference concentration at which differentiation and duplication exactly balance, $p(c_0) = 1/2$, see Figure S6A.

Homeostatic state and its stability

Homeostasis is characterized by a state in which the average stem cell density s and mitogen concentration c take time-independent values. Setting $\dot{s} = 0$ and $\dot{c} = 0$ in Eqs. (1) and (2) and solving for s and c, we find a homeostatic steady state with

$$s_* = \frac{\mu - kc_0}{k'} \ , \qquad c_* = c_0 \ , \tag{4}$$

where c_0 is the reference concentration introduced in Eq. (3). Remarkably, the steady state mitogen concentration c_* is independent of the mitogen production and decay rate. For the steady-state stem cell density s_* to be positive, the condition $\mu > kc_0$ has to be satisfied. For $\mu < kc_0$, the homeostatic state is unstable and all stem cells are lost. In this case, the system attains another type of steady state characterized by complete cell loss, $s = 0$ and $c = \mu/k$. Intuitively, in this case, the mitogen supply is too small to maintain a homeostatic steady state. At $\mu = kc_0$, the system undergoes a transcritical bifurcation where the two steady states exchange their stability.

Whether the system can actually exhibit the homeostatic steady state depends on whether this state is stable or unstable, that is, whether perturbations in the stem cell density or the mitogen concentration decay or grow. To assess the stability of the steady state, we perform a standard linear stability analysis (Strogatz, 1994) and use the ansatz $s(t) = s_* + \xi_s(t)$ and $c(t) = c_* + \xi_c(t)$ in Eqs. (1, 2). Here, ξ_s and ξ_c are small perturbations to the steady state. Linearizing the resulting system in ξ_s and ξ_c, we obtain the governing equations for the perturbations,

$$\dot{\xi}_s = \frac{\Omega\lambda}{k'}\xi_c \ , \qquad \dot{\xi}_c = -(k+\Omega)\xi_c - k'\xi_s \ , \qquad \text{with } \Omega \equiv \frac{m}{2}\frac{s_*}{c_*}k' \ . \tag{5}$$

Combining these equations yields

$$\ddot{\xi}_c + 2\eta\dot{\xi}_c + \omega_0^2\xi_c = 0 \ , \tag{6}$$

where

$$\omega_0 = \sqrt{\Omega\lambda} \qquad \text{and} \qquad \eta = k + \Omega \ . \tag{7}$$

Eq. (6) describes damped harmonic oscillations in the mitogen concentration around its steady-state value, with reference frequency ω_0 and damping coefficient η. Intuitively, when the mitogen concentration is perturbed above its steady-state value, the stem cell density increases due to increased proliferation, thereby increasing the consumption of mitogens, which, in turn, lowers the mitogen concentration, and so on. At the same time, mitogen degradation and consumption provide a damping mechanism which allows the system to relax back to the homeostatic state with a characteristic recovery time η^{-1}. Therefore, perturbations to the homeostatic state will either lead to damped oscillations in the mitogen concentration level (for the case of weak damping, $\eta < \omega_0$) or to oscillation-less decay towards the steady state (for the case of strong damping, $\eta > \omega_0$).

Figures 5D and 5E in the main text show numerical examples of the dynamics generated by Eqs. (1) and (2) for different mitogen production rates μ. Starting from an initial stem cell density below the homeostatic density s_*, the system shows a transient period with decaying oscillations of the cell density and mitogen concentration (as explained above), before settling towards the homeostatic state given by Eqs. (4). These examples also illustrate that the homeostatic stem cell density depends on the mitogen production rate μ, whereas the corresponding mitogen concentration is always $c = c_0$, Eq. (4).

Model with two stem cell compartments

We now extend our mean-field model to include both the stem cell population and the transit amplifying (TA) cell populations, to reflect the observation that not only GFRα1+ cells but also NGN3+ cells, which act as TA cells in this system, consume FGF. This allows us to study whether the additional consumption of mitogens by the latter has a significant effect on the model dynamics. In

this extended model, TA cells arise from stem cells with their differentiation rate $(1-p)\lambda$ and consume mitogens at the same rate as the stem cells. For the time being, we neglect reversion of differentiating cells to the stem cell compartment because it occurs at very low rates during the recovery stages (Hara et al., 2014); however, we will return to this point later. Denoting the average density of TA cells by d, our generalised model is given by

$$\begin{aligned} \dot{s} &= (2p(c)-1)\lambda s \ , \\ \dot{d} &= (1-p(c))\lambda s - \gamma d \ , \\ \dot{c} &= \mu - kc - 2k'p(c)(s+d) \ . \end{aligned} \tag{8}$$

Here, γ is the net loss rate of TA cells through further differentiation. The extended model also exhibits a homeostatic steady state characterized by time-independent values for s, d, and c that satisfy the relations

$$s_* + d_* = \frac{\mu - kc_0}{k'}, \qquad \frac{s_*}{d_*} = \frac{2\gamma}{\lambda}, \qquad c_* = c_0 \ . \tag{9}$$

The first equation implies that the total cell population, composed of both stem cells and TA cells, depends linearly on the mitogen production rate μ. The second equation implies that the ratio of the stem cell and the differentiating cell density is independent of the mitogen production rate μ. Note that the steady state mitogen concentration is the same as for the model with a single stem cell compartment (compare to Eq. (4)).

Figure S6B shows numerical examples of the dynamics generated by Eqs. (8) for different mitogen production rates μ. After an initial transient period, the system settles towards the steady state, characterized by Eq. (9). While the extension of the mean-field model by the dynamics of the differentiating cell population does not significantly alter the phenomenology of the model, it leads to several new predictions characterizing the relation between the two stem cell compartments that can be tested experimentally: While in steady state, the total progenitor cell density $s_* + d_*$ depends on the mitogen production rate μ, while their ratio s_*/d_* and the mitogen concentration c_* do not.

Robustness of the mitogen competition mechanism

Self-renewal by a subpopulation

It is under debate whether all cells have the potential of self-renewal or whether only a subpopulation constitutes the self-renewing compartment (Aloisio et al., 2014; Chan et al., 2014; Tokue et al., 2017). We can allow for the latter scenario in our model as follows: In addition to the TA compartment d, we include two stem cell compartments s and g whose differentiation and duplication rates depend on the mitogen concentration c,

$$\begin{aligned} \dot{s} &= (2p(c)-1)\lambda s \ , \\ \dot{g} &= (2p(c)-1-\varepsilon)\lambda' g + (1-p(c))\lambda s \ , \\ \dot{d} &= (1+\varepsilon-p(c))\lambda' g - \gamma d \ , \\ \dot{c} &= \mu - kc - 2k'p(c)(s+g+d) \ . \end{aligned} \tag{10}$$

Here λ' is the reference duplication/differentiation rate of the g compartment and the parameter ε indicates a tilt of the g compartment towards differentiation—if the g compartment was perfectly

self-renewing, it would grow due to the influx from differentiating stem cells. In this scenario, the cell pool corresponds to $s+g$. The system has a steady state, given by

$$s_*+g_*+d_*=\frac{\mu-kc_0}{k'}\ ,\qquad \frac{s_*+g_*}{d_*}=\frac{\gamma}{1/2+\varepsilon}\left(\frac{1}{\lambda'}+\frac{2\varepsilon}{\lambda}\right)\ ,$$

$$c_*=c_0\ ,\qquad \frac{s_*}{g_*}=\frac{2\varepsilon\lambda'}{\lambda}\ .$$

Importantly, the steady-state mitogen concentration c_* and the total cell density $s_*+g_*+d_*$ retain the same expressions as compared to the previous models, see Eqs. (9). Together with Figure S6C showing a numerical example of the model given by Eqs. (10), these relations show that all key features of the mitogen competition mechanism are preserved: attainment of a homeostatic state, a steady-state mitogen concentration independent of the mitogen supply, linear correlation of the steady-state cell density with mitogen dose, and transient oscillations during recovery.

Dedifferentiation

In the minimal model, Eqs. (8), reversion of differentiating cells to the stem cell compartment has been neglected because it occurs at very low rates during the recovery stages (Hara et al., 2014). To show that, nevertheless, the mitogen competition mechanism is unaffected by such reversion processes, we consider a model in which reversion occurs at a rate $\bar{\gamma}$ as follows,

$$\begin{aligned}\dot{s}&=(2p(c)-1)\lambda s+\bar{\gamma}d\ ,\\ \dot{d}&=(1-p(c))\lambda s-(\gamma+\bar{\gamma})d\ ,\\ \dot{c}&=\mu-kc-2k'p(c)(s+d)\ .\end{aligned}\tag{11}$$

The general expression for the steady-state solution is combinatorially very complex so that, for illustration purposes, we here resort to the case of $m=2$ for the Hill exponent in Eq. (3). In this case, the steady state solution is given by

$$s_*+d_*=\frac{1+(\vartheta_1/\vartheta_2)^2}{2}\frac{\mu-kc_*}{k'}\ ,\qquad \frac{s_*}{d_*}=\frac{\vartheta_1^2+\vartheta_2^2}{\lambda}\ ,\qquad c_*=\frac{\vartheta_1}{\vartheta_2}c_0\ .\tag{12}$$

where the parameters $\vartheta_n=\sqrt{\gamma+n\bar{\gamma}}$ combine the differentiation and dedifferentiation rates γ and $\bar{\gamma}$, respectively, of the differentiating compartment. Despite correction factors in the expressions for s_*+d_*, s_*/d_* and c_*, which account for the presence of reversion, the qualitative key conclusions drawn before remain completely unaltered: the steady-state cell density s_*+d_* depends linearly on the mitogen supply rate μ, whereas the ratio s_*/d_* and the mitogen concentration c_* are independent of the mitogen supply rate μ, decay rate k and consumption rate k'. In addition, Figure S6D shows an example for transient oscillations during recovery in the presence of dedifferentiation.

Stem cell death

In addition to proliferation and differentiation, stem cells may undergo a limited amount of cell death. To illustrate that cell death does not qualitatively alter the features of our model, we consider a variant of the one-compartment model Eqs. (1, 2) including an explicit death term for the stem cells,

$$\dot{s}=(2p(c)-1)\lambda s-\zeta s\ ,\tag{13}$$

where ζ is the death rate. For a non-vanishing death rate, the steady-state stem cell density s_* and

mitogen concentration c_* are perturbed from the corresponding values in the case of no death ($\zeta = 0$),

$$s_* = \frac{\mu - kc_0}{k'(1+\zeta/\lambda)} \quad , \qquad c_* = c_0\left(1 + \frac{2\zeta}{\lambda - \zeta}\right)^{1/m} \quad . \tag{14}$$

For $\zeta = 0$, we recover the previous result Eqs. (4). Importantly, despite correction factors in the expressions for s_* and c_*, which account for the presence of cell death, the qualitative key conclusions drawn before remain completely unaltered: the steady-state stem cell density s_* depends linearly on the mitogen supply rate μ, whereas the mitogen concentration c_* is independent of the mitogen supply rate μ, decay rate k and consumption rate k'.

Secretion of antagonising factors

As noted in the main text, the spermatogenic stem cell compartment might secret one or more factors that antagonize the mitogens. To illustrate the effect of such a factor on the mitogen competition mechanism, we consider the model with a single stem cell compartment, Eqs. (1, 2), and include the dynamics of an antogonizing factor x that is produced by stem cells and causes a reduction of the effect of the mitogen on the cell fate behavior of the stem cells,

$$\begin{aligned} \dot{s} &= (2p(c)r(x) - 1)\lambda s \quad , \\ \dot{x} &= \alpha s - \beta x \quad , \\ \dot{c} &= \mu - kc - 2k'p(c)s \quad . \end{aligned} \tag{15}$$

Here, α is the effective rate with which x is secreted by the stem cells and β is its degradation rate. The duplication probability of the stem cells now depends in a monotonically decreasing way on the concentration of x through the factor $r(x) = (1 + (x/x_0)^m)^{-1}$. Note that for $x = 0$, the system reduces to Eqs. (1) and (2). The above system does not possess a closed analytical expression for the steady state. However, numerical solutions show the existence of such a steady state including damped oscillations during the recovery phase and suggest a nearly linear correlation of the homeostatic cell density with the mitogen dose if the effect of the antagonizing factor is weak, see Figure S6E.

Spatially extended model

To address the effects of localized mitogen source regions, we invoke a spatially extended model that goes beyond the population-level models which only describe the average behavior of the spermatogenic stem cell pool. We replace the total populations and concentrations $s(t)$, $d(t)$ and $c(t)$ by cell density and concentration fields $\phi^S(\boldsymbol{x}, t)$, $\phi^D(\boldsymbol{x}, t)$ and $\phi^C(\boldsymbol{x}, t)$, where $\boldsymbol{x}$ denotes a two-dimensional coordinate that indicates the position on the basement membrane. Because of the tubule topology, we choose cylindrical coordinates $\boldsymbol{x} = (\theta, z)$ where z is the position along the tubule axis and θ is the angular position around the circumference, see sketch in Figure S6F. The simplest hypothesis about the motion of stem cells is a random walk without any preferred direction. The corresponding governing equations retain the interaction structure of the zero-dimensional model, Eqs. (8), but include a diffusion term for the stem cells and a position-dependent source term J for the mitogens,

$$\partial_t \phi^S = \eta \nabla^2 \phi^S + [2p(\phi^C) - 1]\lambda \phi^S \ ,$$
$$\partial_t \phi^D = \eta \nabla^2 \phi^D + [1 - p(\phi^C)]\lambda \phi^S - \gamma \phi^D \ , \tag{16}$$
$$\partial_t \phi^C = \mu J(\theta, z) - k\phi^C - 2k' p(\phi^C)(\phi^S + \phi^D) \ .$$

Here, η is the mobility of stem and progenitor cells, which, for simplicity, we have set to be the same. Since the mitogen sources are most prevalent in the vicinity of the vasculature which runs along the tubule axis, we consider, for simplicity, sources and density fields that are constant along the z-direction, $J = J(\theta)$ and $\phi^S = \phi^S(\theta, t)$, etc. Consequently, terms of the form $\eta \nabla^2 \phi$ simplify to $(\eta/R^2)\partial_\theta^2 \phi$ where R is the radius of the seminiferous tubules. We consider M mitogen sources, with each source $i = 1, \ldots, M$ being centered around an angular position θ_i and having a characteristic angular extension σ, modeled by a normalized Gaussian function $j(\theta) = e^{-\theta^2/2\sigma^2}/\sqrt{2\pi\sigma^2}$. Since the source field J must be a 2π-periodic function, it is given by the cyclic summation of the individual sources,

$$J(\theta) = \sum_{i=1}^{M} \sum_{n=-\infty}^{\infty} j(\theta - \theta_i + 2\pi n) \ . \tag{17}$$

Figures S6F and S6G shows example long-term homeostatic states of the system with a single source ($M = 1$) located around $\theta = \pi$ (panel F) as well as a random distribution of $M = 5$ sources (panel G). Stem and progenitor cells become preferentially localized in the vicinity of the source region. This localization occurs without any explicit chemotactic mechanism; stem cells that randomly move away from the source region tend to differentiate and are therefore lost whereas stem cells that remain in the source region tend to duplicate and thus accumulate. The spatially extended system also retains the major phenomenological features of the population level model: Figure S6H shows the time-dependent line densities s, d and c, defined by

$$s(t) = \int_0^{2\pi} \phi^S(\theta, t) d\theta \quad \text{etc.} \tag{18}$$

for a system with the mitogen source distribution shown in panel G, starting from a small homogeneous stem cell density $\phi^S|_{t=0} = s_0$ and initially no progenitors ($\phi^D|_{t=0} = 0$) and zero mitogen concentration ($\phi^C|_{t=0} = 0$). After exhibiting damped oscillations, the system settles towards a robust homeostatic state with a defined cell density, cf. Figures S6B–S6E.
